# Inhibition of Scavenger Receptor Class B Type 1 (SR-B1) Expression and Activity as a Potential Novel Target to Disrupt Cholesterol Availability in Castration-Resistant Prostate Cancer

**DOI:** 10.3390/pharmaceutics13091509

**Published:** 2021-09-18

**Authors:** Mitali Pandey, Grace Cuddihy, Jacob A. Gordon, Michael E. Cox, Kishor M. Wasan

**Affiliations:** 1 Department of Urological Sciences, Faculty of Medicine, University of British Columbia, Vancouver Prostate Centre, Vancouver, BC V6T 1Z3, Canada; mpandey@prostatecentre.com (M.P.); mcox@prostatecentre.com (M.E.C.); 2 College of Pharmacy and Nutrition, University of Saskatchewan, 104 Clinic Place, Saskatoon, SK S7N 2Z4, Canada; grace.cuddihy417@gmail.com; 3 Oncology Bioscience, Oncology R&D, AstraZeneca, Boston, MA 02451, USA; jacob.gordon@astrazeneca.com

**Keywords:** prostate cancer, castration-resistant prostate cancer, cholesterol uptake, scavenger receptor Class B type I high-density lipoproteins, patient survival

## Abstract

There have been several studies that have linked elevated scavenger receptor class b type 1 (SR-B1) expression and activity to the development and progression of castration-resistant prostate cancer (CRPC). SR-B1 facilitates the influx of cholesterol to the cell from lipoproteins in systemic circulation. This influx of cholesterol may be important for many cellular functions, including the synthesis of androgens. Castration-resistant prostate cancer tumors can synthesize androgens de novo to supplement the loss of exogenous sources often induced by androgen deprivation therapy. Silencing of SR-B1 may impact the ability of prostate cancer cells, particularly those of the castration-resistant state, to maintain the intracellular supply of androgens by removing a supply of cholesterol. SR-B1 expression is elevated in CRPC models and has been linked to poor survival of patients. The overarching belief has been that cholesterol modulation, through either synthesis or uptake inhibition, will impact essential signaling processes, impeding the proliferation of prostate cancer. The reduction in cellular cholesterol availability can impede prostate cancer proliferation through both decreased steroid synthesis and steroid-independent mechanisms, providing a potential therapeutic target for the treatment of prostate cancer. In this article, we discuss and highlight the work on SR-B1 as a potential novel drug target for CRPC management.

## 1. Background and Focus of This Perspective

Prostate cancer (PCa) was the second most frequently diagnosed cancer and the second and fifth leading cause of cancer death among Canadian men and men worldwide, respectively, in 2020 [1]. Androgen deprivation therapy (ADT) is effective for treating PCas that fail primary curative-intent therapies; however, remission is temporary [2,3], and disease re-emergence or castration-resistant prostate cancer (CRPC) occurs in approximately 20% of patients [4,5,6]. Expertly reviewed [7] advances in the understanding of the disease landscape and the mechanisms of progression have resulted in a remarkable array of therapies that have greatly increased the survival of patients with incurable metastatic disease. The focus of this article is on the underappreciated potential of targeting cholesterol metabolism in the advanced disease state and how understanding the role of scavenger receptor class B member 1 (SR-B1) may reveal potential therapeutic opportunities related to cholesterol ester (CE) uptake for de novo steroidogenesis and cellular homeostasis. 

### Methods Used to Review the Literature

This is not a formal “systematic review” article but rather a perspective that discusses the current literature on SR-BI and its potential use as a therapeutic target in treating prostate cancer. Our laboratory is doing extensive research in this area, and this is a perspective that builds on the concepts we are developing around our cited recent publications. The authors relied on their combined breadth of knowledge of the area and used PubMed, Web of Science, and Scopus to complete a thorough scan of the literature.

Androgen receptor (AR) activity is the primary driver of PCa, and its activation persists in the vast majority of CRPC, in part, due to intratumoral androgen synthesis [8,9,10,11]. The recognition that AR signaling remains integral to CRPC drove the development of second-generation AR targeted agents (ARTAs), namely, potent AR ligand-binding antagonists [12,13] and inhibitors of the key enzyme in steroid synthesis from cholesterol precursor, cytochrome P450 isoform 17A1 (CYP17A1) [14,15], that are now routinely used to manage CRPC prior to, or in conjunction with, chemotherapy [16,17]. Results from the LATITUDE and STAMPEDE trials demonstrating that the CYP17A1 inhibitor, abiraterone acetate (Zytiga^®^), improves survival of patients undergoing ADT [18,19,20] indicates the importance of inhibiting CYP17A1, and maximally suppressing steroid synthesis, to manage advanced PCa prior to CRPC progression. Although these, and other non-ARTA therapies, have dramatically improved CRPC management, a substantial fraction of patients either do not respond to ARTAs, or additionally develop resistance to these therapies [21], thus emphasizing the need for novel therapies. 

AR antagonist-resistance is largely achieved by AR amplification and/or mutations in the ligand-binding domain [22,23]; AR splice variants [24,25,26,27]; alterations in the AR, DNA repair, cell cycle, and phosphatase; Tensin Homolog deletion on chromosome 10 (PTEN); phosphatidylinositol 3 kinase-Ak strain-transforming mammalian target of rapamycin (PI3K-AKT-mTOR and wingless-related integration of site-β catenin (Wnt-β-catenin) pathways [28]; or progression to a spectrum of “androgen-indifferent” amphicrine, double-negative, and neuroendocrine PCa (NEPC) phenotypes [29,30]. Resistance to androgen synthesis inhibitors is thought to be due to the amplification of enzymes, namely, CYP17A1, which result in intratumoral accumulation of higher-order steroids [28]. Abiraterone-resistant CRPCs also gain CYP17A1-independence by gaining AR mutants that are responsive to steroid precursors such as pregnenolone and progesterone [31,32]. While these mechanisms lead to AR promiscuity and hypersensitivity [33,34], they do not impede the response to steroid precursors [35,36,37,38,39,40].

Cholesterol is the essential precursor of steroid synthesis, including androgens [41]. AR signaling drives PCa and plays an essential role in the development and progression of the disease [42,43,44,45,46,47]. As an essential component in cell membranes [48], and a mediator of cell proliferation and inflammatory pathways [41,49], there are multiple ways cholesterol needs can be exploited to target PCa pathophysiology. Fortunately, this has led to investigations into potential therapies that either directly or indirectly affect cholesterol, which could potentially impact PCa progression. Cholesterol can either be taken up from circulation, or synthesized de novo, and serves numerous critical functions in mammalian cells. One of the ways in which cells take up cholesterol/CEs from the circulation is through cell surface receptor SR-B1 [50]. This perspective highlights and discusses research surrounding the role of cholesterol in disease progression and the potential of SR-B1 as a potential novel therapeutic target for the management of CRPC.

## 2. Cholesterol

### Lipoproteins and Transport

**Figure 1:**
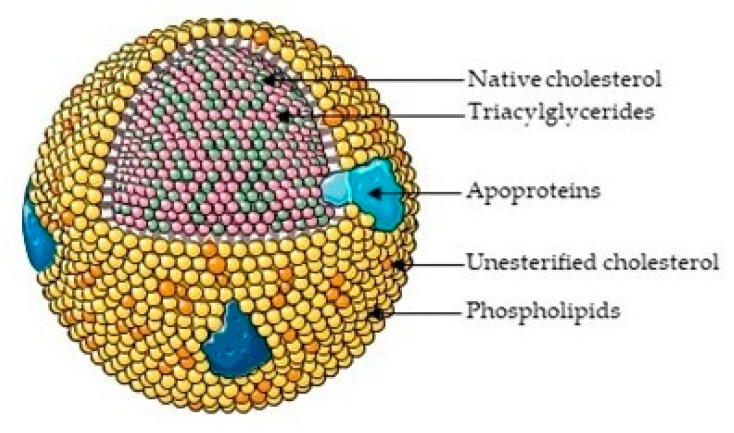
Lipoprotein structure—an overview. Lipoproteins are a spherical monolayer of amphipatic phospholipids (yellow) and free/unesterified cholesterol (orange). The tails of the phospholipids create a hydrophobic core of non-polar lipids, primarily cholesterol esters (green) and triglycerides (pink), surrounded by a hydrophilic membrane of phospholipids, free cholesterol and apolipoproteins (blue). Lipoproteins differ in their lipid composition, size, density, major apolipoproteins and function [53]. Figure produced using Servier medical art [54].

Cholesterol is a multifunctional non-essential nutrient, which is important as a component of cell membranes and as a precursor of bile acids, vitamin D, and steroid hormones [51]. Cholesterol is synthesized primarily by the liver through the mevalonate pathway and then transported through the vasculature complexed within single, phospholipid membrane structures known as lipoproteins. Apolipoproteins are proteins that bind lipids and fat-soluble vitamins to form lipoproteins [52]. Different lipids present on apolipoproteins determine the functional identity of lipoproteins and their subsequent role within the human body. Cholesterol can either be present on the hydrophilic surface of the lipoprotein as native cholesterol or within the hydrophobic core as CEs (Figure 1) [53].

**Figure 2:**
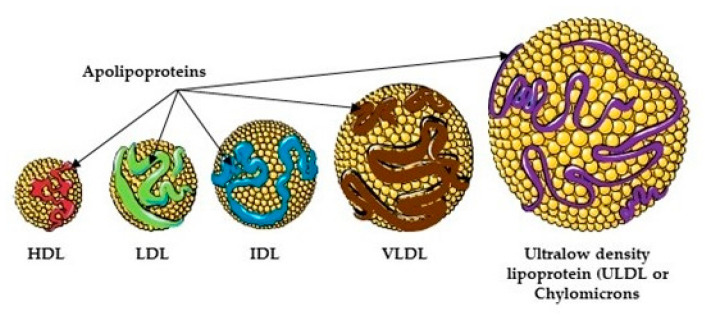
Lipoprotein—types and function. Four kinds of lipoproteins exist in the human body *viz.* high density lipoprotein (HDL), low density lipoprotein (LDL), very low density lipoprotein (VLDL) and one intermediate product, intermediate density lipoprotein. Nomenclature is based on the ratio of their size and density (g/dL): HDL (7–13 nm in diameter, 1.063–1.25), LDL (22–27 nm, 1.019–1.063), ILDL (27–30 nm, 1.006–1.019), VLDL (35–80 nm, 0.95–1.006), chylomicrons (80–1200 nm, <0.95). Their function is determined by the apolipoproteins on their surface. Major apolipoproteins present on lipoproteins include A-I, A-II, C, E in HDL (orange), B-100 in LDL (green); B-100, C, E in LDL (blue), and VLDL (brown) and B-48, A-I, A-II, C, E in chylomicrons (purple). HDL removed cholesterol from peripheral tissue, LDL delivers fat to peripheral tissue, IDL and VLDL transport fats from the liver, while chylomicrons transport dietary fats (food and bile). Figure produced using Servier medical art [54].

Lipoproteins are classified by the inverse relationship of size/density, i.e., larger and lower density and smaller and higher density [53]. Lipoproteins are large and less dense when the fat to protein ratio is increased. They are most commonly classified as high-density lipoproteins (HDL), low-density lipoproteins (LDL), intermediate-density lipoproteins, very-low-density lipoproteins (VLDL), and ultra-low-density lipoproteins (ULDL), which are also known as chylomicrons (Figure 2). Each lipoprotein type carries out a specific function and is associated with a different disease [55]. HDL collects fat molecules from cells and tissues and takes them back to the liver; LDL carries 3000–6000 fat molecules around the body; VLDL carries newly synthesized triacylglycerides from the liver to the adipose tissue; and chylomicrons carry fat from the intestines to the liver, skeletal muscle, and adipose tissue [53].

**Figure 3:**
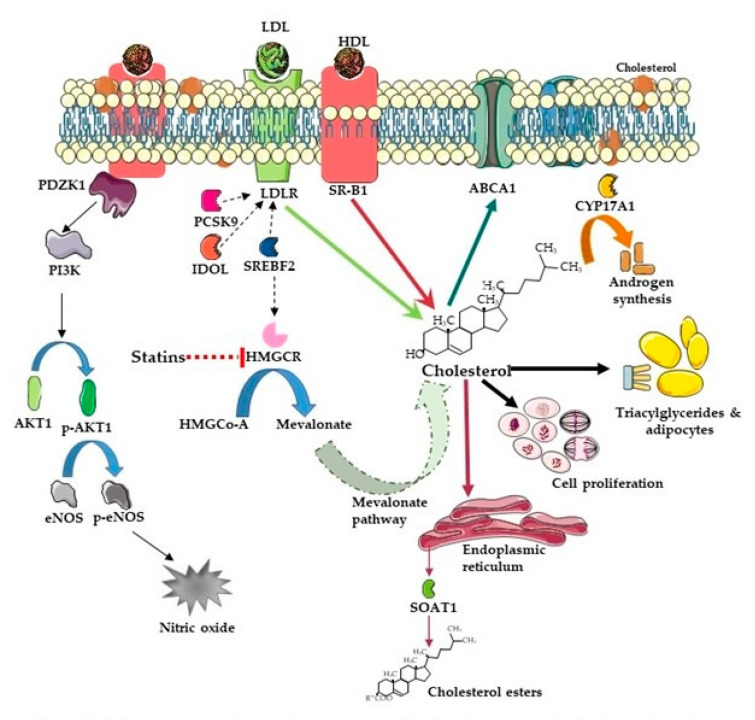
Cholesterol–intracellular and intratumoral metabolism. Cellular uptake of cholesterol is mediated by endocytosis of low density lipoproteins (LDL) via the LDL receptors (LDLRs), and partially trough HDL receptors (HDLRs), ore commonly known as scavenger receptor, Class B1 (SR-B1). LDLR levels in cells is tightly regulated by proprotein convertase subtilisin/kexin type 9 (PCSK9) and inducible degrader of LDLRs (IDOL). In endothelial cells, HDL is a chemotactic factor and signaling is mediated SR-B1 which is associated with (PDZ Domain Containing 1 (PDZK1) and sphingosine phosphate (S1P) receptor 1 (S1PR1) a scaffolding protein, leading to phosphorylation of AKT1 (p-AKT1) which phosphorylates and activates nitric oxide synthase to produce nitric oxide (NO). Cholesterol can also be produced de novo in cells via the mevalonate pathway. Reduction of 3-hydroxy-3-methyl-glutaryl-CoA (HMG-CoA) to mevalonate is controlled by 3-hydroxy-3-methyl-glutaryl-coenzyme A reductase (HMGCR), the enzyme targeted by statin drugs. Cholesterol efflux is facilitated by ATP Binding Cassette Subfamily A Member 1 (ABCA1) transporter. Once in the cell, cholesterol is involved in multiple functions including cell proliferation and androgen synthesis. Excess intracellular cholesterol can get esterified to cholesterol esters by sterol *O*-acyltransferase 1 (SOAT1) and stored as lipid droplets. Broken and continuous arrows indicate regulation and metabolic steps respectively. Figure produced using Servier medical art [54].

Cholesterol is also obtained from diet and bile, where it is taken up from the intestine by enterocytes. Here, it is packaged into the triacylglyceride-rich chylomicrons or VLDL that also contain cholesterol and apolipoprotein B-48 (apo B-48), a truncated form of apolipoprotein B (apo B). Chylomicrons then enter the lacteals and the bloodstream through the thoracic duct [53]. The triglyceride contents of chylomicrons are metabolized by lipoprotein, lipase and the resultant high cholesterol chylomicron remnants are then endocytosed by the liver. VLDL, secreted from the liver, contains high levels of triglyceride complexed with apolipoprotein B-100 (apo B-100). It is this full form of LDL that is recognized by the LDL receptor (LDLR). The triglycerides within these particles are similarly metabolized by lipoprotein lipase. This results in smaller particles sometimes referred to as intermediate density lipoprotein (IDL) and eventually into cholesterol containing LDL [53,56]. Oxidation of either the lipid or apolipoprotein constituents of LDL leads to conformational changes, which prevent its uptake via LDLR; instead, it is taken up by scavenger receptors [57]. The reverse role in cholesterol transport, where cholesterol is transferred from peripheral tissues to the liver, is regulated by apolipoprotein A-I (Apo A-I), which is the major component of HDL [56,58]. HDL produced by the liver receives cholesterol through apo A-I’s interaction with the ATP-binding cassette transporter ABCA1 or the cholesterol efflux regulatory protein (CERP) in peripheral tissues and releases cholesterol to tissues expressing SR-B1 Figure 3 [59]. It is HDL whose major component is apo A-I [60,61,62].

#### Cholesterol and Prostate Cancer (PCa)

Cholesterol was first observed as crystals present within tumors in 1909 [63], and increased cholesterol levels were first reported in prostate tumors in 1942 [64]. The subsequent wealth of epidemiological evidence regarding cholesterol levels in PCa produced confounding results. Several studies reported that lowering cholesterol increased cancer incidence [65,66], while others found no association between cholesterol and cancer [67]. A meta-analysis of the relationship between circulating cholesterol and PCa was conducted in 52 population studies, of which 32 studies found an inverse association between cancer risk and cholesterol level [68]. The authors also concluded that there was no absolute level of cholesterol associated with cancer; rather, the low cholesterol cohort, relative to the cohort average in any population, had a greater prevalence of cancer.

In contrast, men with high blood-cholesterol levels have been reported to be at an increased risk of PCa and PCa mortality [69,70,71]. Furthermore, cholesterol levels have been shown to be elevated in post-ADT patient serum [72,73,74,75,76,77,78,79] and bone metastasis [80,81]. Investigations into alterations specific to cholesterol metabolism within the tumor setting have generated evidence that hypercholesterolemia was associated with an increase in tumor volume, progression, and incidence in the transgenic adenocarcinoma mouse prostate (TRAMP) [82] and increased tumor growth, AKT activation, and intratumoral androgens in xenograft models [83,84,85]. Thus, there is emerging evidence that implicates a positive association between cholesterol and PCa [86].

## 3. Intratumoral Androgen Synthesis in Castrate-Resistant Prostate Cancer

As stated earlier, AR activity persists in the majority of CRPCs, and it is presumed that progression to CRPC is driven by reactivated AR activity despite patients being on ADT and having castrate androgen levels. Steroidogenic CRPCs rely on cholesterol for androgen biosynthesis and prostate tumors have increased levels of intracellular cholesterol precursors [87,88]. This demand for cholesterol in cancer cells is met by uptake from blood and de novo cholesterol synthesis. Systemic cholesterol, predominantly found and transferred by lipoproteins, is taken up by cancer cells through the actions of LDLR [89] and scavenger receptors [90]. Upregulated expression of HMGCR, LDLR, SR-B1, sterol O-acyltransferase1 (SOAT1), the enzyme that catalyzes the reaction between long-chain fatty acids and intracellular cholesterol to form hydrophobic CE [91], and loss of the function of ATP-binding cassette transporter A1 (ABCA1), which mediates cholesterol efflux from cells, further facilitates enrichment of cellular cholesterol pools (Figure 3) [92,93,94,95]. Many of these key nodes in cholesterol metabolism are drug targets, which might be used to modulate intratumoral cholesterol metabolism. For instance, proprotein convertase subtilisin/kexin type-9 (PCSK9), a serine protease, modulates cholesterol metabolism by binding to LDLR and targeting it for lysosomal destruction [96] (Figure 3). It is also involved in degradation of other LDLR family members, namely VLDLR, lipoprotein receptor-related protein 1 (LRP-1), and apolipoprotein E receptor 2 (apo ER2) [97]. PCSK9 inhibitors represent a new class of drugs for treatment of CRPC in patients with familial hypercholesterolemia who do not respond to statins [97,98].

Adrenal androgens have long been considered a source of ligands for AR reactivation [99]; however, it is now appreciated that elevated intratumoral androgen levels in CRPC [9] can be explained by de novo intratumoral androgen synthesis as evidenced by elevated expression of steroidogenic enzymes essential for conversion of the precursor, cholesterol, into androgens at levels sufficient to activate the AR [8,10]. These integral points in androgen biosynthesis must be explored for novel therapies in order to improve outcomes in steroidogenic CRPC. Thus, disrupting the ability of PCa to synthesize or sequester sufficient cholesterol for steroidogenic and biogenic needs is a viable therapeutic opportunity for management of CRPC. Table 1 summarizes therapies that have been developed due to their effect on cholesterol metabolism.

## 4. Effect of a Class of Drugs That Inhibit Cholesterol Synthesis on Prostate Cancer (PCa)

The seminal discovery that a class of drugs, called “statins,” are efficient inhibitors of de novo cholesterol synthesis dramatically altered cardiovascular disease mitigation [105,106,107,108]. Similar in structure to HMG-CoA, statins fit into the enzymatic active site of HMG-CoA reductase to compete with native HMG-CoA binding [109,110]. This competition reduces the rate at which mevalonate is produced, thus inhibiting production of cholesterol in the liver [111]. In addition, statins also affect systemic cholesterol availability by promoting a compensatory decrease in circulating LDL and cholesterol transport through a variety of mechanisms [93,112,113]. The broad use of statins as first-line therapeutics for prevention of cardiovascular disease by inhibition of cholesterol synthesis raised the possibility of their effect on PCa. Findings from several studies indicate that statins may also play a role as anti-cancer agents in PCa via both cholesterol and pleiotropic non-cholesterol-mediated effects [108,114,115,116,117,118]. In preclinical settings, simvastatin and lovastatin have been found to reduce cell viability through an induction of apoptosis; to inhibit cell proliferation, colony formation, and migration; to suppress the AKT pathway; and to cause G_1_ phase cell cycle arrestation in AR-positive LNCaP, AR-negative PC-3, and DU145 cells, while atorvastatin, simvastatin, and rosuvastatin have all been shown to reduce the metastatic potential of PC-3 cells [119,120,121,122,123,124,125,126,127,128].

From an epidemiological perspective, several attempts have been made to understand the effect of statins on both the probability of occurrence of PCa and the clinical outcomes of those diagnosed with PCa [116]. While PCa epidemiologists increasingly correlate statin use with decreased PCa occurrence, and improved disease prognosis [129,130,131,132,133,134,135,136,137,138,139,140,141,142,143,144,145,146,147,148,149,150,151,152,153,154], there exists a vast body of literature surrounding the total incidence of PCa with conflicting results. Several studies have reported no effect on the incidence of PCa with statin use [107,155,156,157,158,159,160,161,162,163,164,165,166], and one study associates statin use with an increased incidence of PCa [167]. These studies have been summarized in Table 2.

Despite the uncertainty as to whether statins impact overall PCa incidence, evidence that statin use may reduce the incidence of advanced PCa continues to grow. In an anecdotal case, a patient with bone metastatic CRPC who started on abiraterone and prednisolone, and soon after on atorvastatin, achieved a complete PSA response with no evidence of bone metastasis in six months [169]. Reports indicating that statins improve PSA decline and overall survival of abiraterone-treated patients [170,171] corroborate observations that statin use significantly increases progression-free, and overall, survival of abiraterone-treated CPRC patients [172]. These clinical results support previous conclusions that this is, in part, due to a decrease in de novo cholesterol and androgen synthesis [126,173,174,175]. Although not without contradiction, the wealth of evidence including consistent preclinical findings suggest that all elements of cholesterol homeostasis are deregulated in PCa to promote cellular cholesterol accumulation. This demonstrates the possibility of targeting cholesterol metabolism, through statins or potentially through other cholesterol metabolism proteins, as therapeutic targets in the treatment of PCa.

## 5. SR-B1

Scavenger receptors are a diverse group of sequence-unrelated proteins united by their ability to recognize common polyionic ligands [176]. Class B is comprised of three members whose short N- and C-terminal cytosolic regions regulate signal transduction and trafficking [176]. SR-B1 is encoded by the *SCARB1* gene and is a highly glycosylated, ~82 kDa cell-surface-receptor protein that contains two transmembrane domains located near to its cytosolic *N*- and *C*-terminal domains [177,178,179,180].

### 5.1. Transcriptional Regulation

Similar to LDLR, SR-B1 is ubiquitously expressed, but shows particularly high expression in the liver as well as steroidogenic tissues including the adrenal glands, ovaries, and testes [181,182]. It is differentially regulated between the liver and steroidogenic tissues. In steroidogenic cells, SR-B1 is primarily regulated by trophic hormones such as luteinizing hormone (LH), follicle stimulating hormone (FSH), and adrenocorticotropic hormone (ACTH) [177,178,182,183,184,185]. Its expression in liver cells, however, can be affected by both hormonal control as well as dietary fats and pharmacologic agents, notably fibrates [182,186]. Prolonged adrenal stimulation by adrenocorticotropic hormone (ACTH) has been shown to induce SR-B1 expression in adrenocortical cells and reduce hepatic expression in mice and rats, suggesting a mechanism for the preferential channeling of cholesterol to the adrenal tissue [178,187,188]. On a cellular level, SR-B1 has been shown to be regulated in a rather complex manner including generic transcription factor binding sites for sterol regulatory element-binding proteins (SREBPs), steroidogenic factor-1 (SF-1), Liver X receptors (LXRs), liver receptor homolog 1, and others [181,182,189,190]. Evidence demonstrates that SR-B1 regulation by trophic hormones may be dependent on cAMP/protein kinase A (PKA) signaling stimulating transcriptional function of steroidogenic factor 1 (SF-1) and CCAAT-enhancer-binding proteins (C/EBP) [191,192]. Cholesterol regulation of SR-B1 has also been demonstrated through SREBP and LXR functions implicating that multiple signaling pathways participate in SR-B1 expression [182]. Its allelic variants are linked to an increased risk of atherosclerosis [193], infertility [194], and/or an impaired innate immune response [195,196,197].

### 5.2. SR-B1 Function

SR-B1 is primarily involved in the selective uptake of CEs from circulating HDL acetylated LDL (AcLDL) and oxidized LDL (OxLDL) [198] and binding to certain viruses and bacteria by a non-endocytic process [199,200,201]. The mechanism of HDL/SR-B1-mediated cholesterol uptake is not fully understood. Unlike LDLR-mediated LDL uptake, SR-B1 does not degrade HDL, rather it mediates selective CE uptake from HDL, AcLDL, and OxLDL in the absence of holo-lipoprotein uptake: that is, without the uptake and lysosomal degradation of the HDL particle itself [202,203]. This appears to be facilitated either by a “non-aqueous” channel passing through the protein [198] or by lipoprotein internalization after binding SR-B1 in a non-clathrin-coated vesicle manner, after which the CE-depleted lipoproteins are secreted [204,205]. It has been proposed that SR-B1-mediated CE uptake is a three-step process. First, a hydrophobic tunnel is formed by the extracellular domain of the receptor between the lipoprotein particle and the cell membrane. CE diffuse through this tunnel into the cell in a concentration gradient manner [206]. The high-resolution crystal structure of the extracellular domain of LIMP-2, a homologue of SR-B1, further corroborates the validity of this mechanism [207].

SR-B1 also facilitates the efflux of free cholesterol between cells and lipoproteins [208]. This mechanism, known as reverse cholesterol transport (RCT), consists of the transport of cholesterol via HDL from peripheral tissues such as macrophages or endothelial cells to the liver where this is utilized for bile acid production, cholesterol excretion, and steroidogenic organs [180,209]. This SR-B1-mediated cholesterol uptake by steroidogenic tissues [210] is critical for androgen synthesis and for the growth and survival signaling of non-steroidogenic endothelial cells [211,212,213]. In endothelial cells, SR-B1 is critical for HDL-induced activation of endothelial nitric oxide synthase (eNOS) leading to, at least in part, the athero-protective effects associated with HDL [214,215,216,217] (Figure 3.). The activation of eNOS, in turn, leads to increased concentrations of nitric oxide (NO) known to have a number of positive effects on cardiovascular health including vasodilation, endothelial regeneration, inhibition of leukocyte chemotaxis [218,219], and prevention of SR-B1-mediated induction of apoptosis [214,220]. Several pathways have been identified as potential mechanisms for this SR-B1-dependent signaling. HDL is known to carry several lipids beyond cholesterol, including lipid-soluble vitamins and sphingolipids [214]. Although insufficient on its own, sphingosine-1-phosphate (Sp1P), when associated with HDL, has been shown to be involved in the activation of eNOS [214,221,222,223]. The mechanism by which Sp1P or other SR-B1 inducers drive eNOS activation is believed to be through the PI3K signaling pathway [214]. HDL binding to SR-B1 has been shown to lead to the activation of the non-receptor tyrosine kinase, c-Src, via the scaffolding protein PDZ domain-containing 1 (PDZK1) that is associated with SR-B1, which, in turn, activates both the PI3K/AKT and the RAS/ERK1/2 pathways, resulting in eNOS activation through serine 1179 phosphorylation [211,212]. Although the relationship between SR-B1 and these signaling pathways has largely only been established in endothelial cells, aberrations in the PI3K pathway and the RAS pathway have been identified and implicated in advanced PCa and highlight the importance of their consideration when studying SR-B1 in this disease context [224]. In macrophages, SR-B1 is associated with a reduction in atherosclerosis [225].

**Figure 4:**
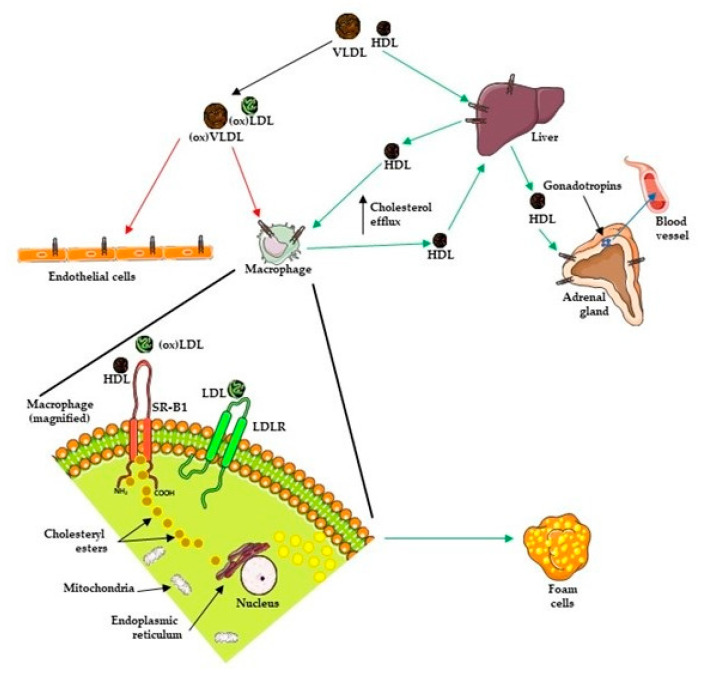
Role of SR-B1 in the development of foam cells from macrophages. Modified lipoproteins are taken by *via* SR-B1 present on macrophage. Increased uptake of these modified lipoproteins and cholesterol esters lead to excessive fat in cells, culminating in the formation of foam cells, the main constituents of atherosclerotic lesions. Figure produced using Servier medical art [54].

SR-B1 has been associated with a reduced risk of developing atherosclerosis. In macrophages, SR-B1 functions in the uptake of modified lipoproteins as well as in secreting cholesterol to HDL. Macrophages are incapable of limiting their uptake of modified lipoproteins via SRs and depend on cholesterol efflux mechanisms via SR-B1 for maintaining cholesterol homeostasis within the cell. Macrophage apoptosis and efferocytosis are key determinants of atherosclerotic plaque inflammation and necrosis. In macrophage-deficient mice, SR-BI promotes defective efferocytosis signaling via the c-Src/PI3K/Rac1 pathway, resulting in increased plaque size, necrosis, and inflammation [226]. Macrophage-derived foam cells, which develop as a result of excessive accumulation of lipoprotein-derived cholesterol [227,228,229,230], play an important role in all stages of atherosclerotic lesion development [231]. In early atherosclerotic lesion, it is these macrophage-derived foam cells that are the predominant constituent of the fatty streak. In advanced atherosclerotic lesions, foam cells are detected as clusters of cells surrounding a core of lipid and necrotic material, where they modulate the stability of the atherosclerotic lesion (Figure 4).

### 5.3. Inhibitors of SR-B1

SR-B1 mediates the selective uptake of CE from HDL into cells and the efflux of cholesterol from cells to lipoproteins. The need to understand the mechanism behind the process, which is distinct from the endocytoic uptake of other lipoproteins, led to the characterization of small molecules that could modulate SR-B1 function. 

Blocker of lipid transfer (BLT) *viz*. **BLT-1 through 5**, a family of thiosemicarbazone copper chelators, were the first inhibitors to be characterized that could inhibit both cellular selective lipid uptake of HDL CEs and efflux of cellular cholesterol to HDL [232,233]. The inhibitory effects of the BLTs were specific to SR-B1 and did not interfere with several clathrin-dependent and -independent endocytic pathways, the secretory pathway, or the actin or tubulin cytoskeletal networks [233]. Of these five compounds, BLT-1 covalently and irreversibly bound to cysteine 384 of SR-B1 [234] in nanomolar concentrations to block CE influx and cholesterol efflux, while BLT-4 was demonstrated to specifically block both SR-B1-mediated influx of CEs and ABCA1-mediated cholesterol efflux to lipid-poor apo A-I [235]. However, in spite of its specificity and nanomolar potency, BLT-1 is extremely toxic to cells, which has limited its use to short-term in vitro assays.

**Glyburide**, a sulfonylurea thought to be capable of binding to SR-B1, was not specific to it; rather, it bound to and inhibited sulphonylurea receptors 1 and 2 (SUR1 and SUR2), members of the ABC superfamily of proteins [235].

**ITX-5061**, an arylketoamide derivative and initially characterized as a type II inhibitor of p38 MAPK, is also an SR-B1 antagonist and a potent SR-B1-mediated hepatitis C virus (HCV) entry inhibitor. In animal models, ITX-5061 was shown to increase serum HDL and apo A-I levels without affecting LDL/VLDL levels [236]. However, phase I clinical trials with ITX-5061 in liver transplant recipients with HCV were terminated [237], [238].

**R-138329** increases plasma HDL cholesterol via inhibition of SR-BI-mediated selective lipid uptake [239]. In vivo studies on mice indicate R-138329 is principally involved in the inhibition of SR-BI-mediated selective lipid uptake in the liver.

High-throughput screening of the National Institutes of Health Molecular Libraries Probe Production Centers Network compound library led to the discovery of a potent class of indolinyl-thiazole-based inhibitor of SR-B1 *viz*. **ML278** [240,241], a bisamide-heterocycle inhibitors of SR-B1 *viz*. **ML279** [241,242] and 8-membered benzo-fused lactam *viz*. **ML312** [241,243]. All three compounds are low nanomolar, reversible inhibitors of SR-B1 with improved potency (ML312 > ML279 > ML278), decreased toxicity liabilities, and the capability to inhibit both SR-BI-mediated lipid uptake and the efflux of free cholesterol to HDL particles [241].

Nanoparticle mimetics of HDL and apo A-II have been demonstrated to be effective targets in nasopharyngeal carcinoma [244] and in pancreatic cancers, respectively [245]. SR-B1 is up-regulated in both cancers. Thus, biomimicry may be an important role in the next generation of cancer therapies. [246]. While these approaches to generate inhibitors of SR-B1 have been successful in vitro and some in vivo models, there still exists the need to develop an inhibitor that can achieve clinical utility.

### 5.4. SR-B1 and PCa

Several lines of evidence link elevated SR-B1 to PCa aggressiveness. SR-B1 expression is correlated with elevated expression of the androgen synthesis enzymes, 3β-hydroxysteroid dehydrogenase (3β-HSD), 17β-HSD, and the mammalian target of rapamycin (mTOR) complex 1 target, ribosomal protein S6 [247]. Pre-clinically, elevated SR-B1 expression is observed in CRPC derivatives of LNCaP, an androgen-responsive PCa cell line, [248,249], and with Western-diet-induced tumor development in the TRAMP model [81]. siRNA silencing of SR-B1 has been shown to decrease PSA secretion and cell viability in C4-2, an LNCaP-derived CRPC model [249]. We and others have demonstrated that, clinically, elevated SR-B1 expression has been found to be associated with high Gleason-grade primary PCa wherein high expression was correlated with decreased disease-specific survival of PCa patients [90,250]. Its transcript levels were elevated in PCa versus benign prostate, as well as in tumors that failed androgen receptor pathway inhibitor (ARPI) therapy and progressed and in NEPC versus CRPC. Overall, the aforementioned findings underlie the apparent potential in targeting cholesterol metabolism as a mechanism for impeding proliferation of PCa and implicating SR-B1 as an actionable target for managing CRPC. A number of studies have already investigated SR-B1 as a potential therapeutic target in CRPC—these findings are summarized in Table 3.

## 6. Stress and Autophagy

AR activity is a critical driver of metabolic reprogramming in PCa [253,254]. AR-mediated enhancement of oxidative phosphorylation and anabolic activity are necessary to sustain the increased demand for the synthesis of lipids, nucleotides, and amino acids [255]. ARTAs suppress these AR-driven metabolic events and activate acute stress pathways to sustain tumor viability. Increased expression of pro-survival chaperones, such as heat shock protein (HSP) 27 and clusterin (CLU) promote pro-survival and anti-apoptotic signaling events [256,257,258,259], enhance metastatic capacity [260], and suppress mitotic activity as part of chemotherapy resistance [261]. CLU enhances PCa survival by preventing protein aggregation and inducing autophagy: an important pro-survival nutrient recycling stress response [248,262]. In this context, it is notable that SR-B1-antagonized PCa cells significantly upregulate CLU and autophagy [250].

Autophagy functions as a robust mechanism by which PCa copes with several cellular stresses including ADT, chemotherapy, and nutrient deprivation, which would otherwise be lethal [263,264,265,266]. Autophagy is generally considered to be primarily regulated by mTOR through AMP-activated protein kinase (AMPK) activation due to low glucose and ATP [267]. There appears to be a stark induction of autophagy in SR-B1-antagonized cells [250]. Although the initial results indicated the role of reduced mTOR signaling as a driver of autophagic phenotype, further characterization of the mechanism of the observed stress remain to be investigated. These include downstream mTOR signaling pathways including S6 Kinase 1 (S6K1), the eIF-4E binding proteins (4E-BP1/2), or Unc-51-like autophagy activating kinase (ULK1/2) through which mTOR mediated signaling drives its regulatory effects [268].

External to mTOR–regulated autophagy, AMPK is a critical energy/nutrient regulatory protein. It functions as a sensor for depleted ATP conditions within the cell promoting catabolic processes to generate more ATP, which have been connected to metabolic processes including autophagy [269]. Capable of activating autophagy through both ULK1 phosphorylation and mTOR, inhibition, AMPK activity has been shown to be reduced by androgen-driven reduced expression of liver kinase B1 (LKB1) and may play a role in SR-B1 driven autophagy [269,270]. Several genes involved in AMPK signaling in androgen-dependent PCa cell lines are involved in lipid metabolism. These include HMGCR, fatty acid synthase (FASN), and 6-phosphofructo-2-kinase/fructose-2,6-biphosphatase 2 (PFKFB2). Inhibiting AMPK signaling using BAY-3827 resulted in downregulation of lipase E (LIPE), cAMP-dependent protein kinase type II-beta regulatory subunit (PRKAR2B), and serine-threonine kinase AKT3 in these PCa cell lines. In addition, BAY-3827 inhibited expression of members of the carnitine palmitoyl-transferase 1 (CPT1) family, which was paralleled by impaired lipid flux [271].

Autophagy is also induced independently of mTOR, through endoplasmic reticulum stress (ERS)-induced expression of chaperones such as binding immunoglobulin protein (BiP), along with inositol-requiring enzyme 1 alpha (IRE1α) through the unconventional splicing x-box protein 1 (XBP1) [272,273,274,275]. Altered cholesterol metabolism is also known to induce ERS mechanisms, and disrupted lipid equilibrium induces the unfolded protein response (UPR) [276]. The UPR, generally in response to an accumulation of misfolded proteins, activates adaptive pathways that attenuate general protein translation, increase molecular chaperone expression, and induce cell cycle arrest to alleviate the ERS [276]. SR-B1 antagonism with either siRNA knockdown or BLT-1 treatment suppresses mTOR activity and induces BiP and, to a lesser extent, IREα expression [250]. Taken as a whole, these results indicate that SR-B1 antagonism induces a strong stress response integrating autophagic and ERS pathways.

## 7. Future Perspectives

CRPC requires androgens for its development. Initial ADT on primary PCa puts selection stress on the cells to develop new methods to generate their own AR. One of the ways PCa cells do this is by initiating de novo cholesterol synthesis and enhanced expression of SR-B1. To address low blood cholesterol, PCa cells increase the expression of SR-B1 to enhance the amount of cholesterol being received by cells. This perspective provides insight on targeting cholesterol metabolism in PCa by regulating SR-B1. Analysis of the relationship between SR-B1 expression at disease onset with patient outcomes could be performed using histological methods on tissue biorepository specimens powered to identify biomarkers for recurrence-free survival [277,278,279,280]. Such repositories consist of radical prostatectomy-derived specimens and corresponding patient follow-up allowing for direct comparison of protein expression to clinical outcome. This would allow for the analysis of the role of cholesterol metabolism proteins in biochemical recurrence as well as assessment of expression across the disease state [281].

Previous studies have highlighted the results of targeting SR-B1 in well-established human and animal PCa models [250,252]. However, determining the expression of SR-B1 and the effects of targeting it in additional AR-expressing PCa models, including the AR-driven but androgen-independent cell lines LN-AI, LN95, and 22Rv1, could provide additional insight into the role of SR-B1 in further progression of the disease. Since androgen independence of these lines is attributed to increased ligand binding domain-deficient AR splice variant, AR-V7, these models would allow interrogation of disease states that are not dependent on cholesterol for de novo steroidogenesis but that are AR-driven [282]. The apparent increased expression of SR-B1 in NEPC mRNA datasets and the sensitivity of the “double negative” PCa models, PC3 and DU145, to SR-B1 antagonism [250,252] provides an interesting fundamental basis for further investigation for a role of SR-B1 in the emergence and proliferation of “androgen-indifferent” PCa. NEPC model H660 and enzalutamide-resistant LNCaP-derived 42D and 42F cells [283,284] can be used as both a direct test of the transcript predictions of increased SR-B1 expression in NEPC and as a model for analyzing SR-B1 antagonism in NEPC. A critical question is whether SR-B1 antagonism can restore sensitivity to ARTAs such as enzalutamide in treatment-resistant disease. Moreover, PCa cells that maintain steroidogenic potential and AR expression such as VCaP would provide further context to effects on steroidogenic potential [285].

Although the use of immortalized PCa cell lines provides valuable tools for evaluating potential therapeutic targets, these models are limited by selective pressures including adaptions to continued proliferation in a non-tumor environment, leading to a poor correlation with clinical outcomes [286]. The advent of patient-derived xenografts maintained in murine hosts provides a powerful approach to evaluating therapeutic potential across the heterogeneity of the disease. With the caveat that mice are HDL-dominant, xenograft tumors, directly implanted from patient to mouse, are thought to better replicate clinical PCa, avoiding the genetic drift observed within immortalized cell lines [287,288,289,290]. Examining the effects of SR-B1 antagonism in either the cell lines or patient-derived xenografts would provide insight into the role of SR-B1 in PCa and which PCa phenotype is most impacted by its antagonism. A broad survey of models would be invaluable for efforts to determine the mechanism driving increased SR-B1 expression across the phenotypic spectrum of lethal CRPC.

Beyond our comprehension of how SR-B1 antagonism may be most effective, additional investigation into the mechanism of the anti-proliferative effects is warranted with respect to both cholesterol synthesis and cholesterol uptake inhibition. The findings discussed in this perspective describe the ability to inhibit both PCa growth and progression by limiting of cholesterol availability. The inability of exogenous steroids to revert the effects of SR-B1 antagonism in AR-responsive models, and the efficacy of SR-B1 antagonism to suppress the growth of AR-null PCa models, suggests that reducing cholesterol availability, at least by SR-B1 antagonism, is not primarily responsible for anti-tumor activity [250]. This finding emphasized the impact of more general nutrient-deprived stress mechanisms driven because of impacted cholesterol accumulation. To adapt to cellular stresses such as nutrient deprivation, both cancerous and non-cancerous cells employ several mechanisms of adaptation allowing for continued survival in harsh tumor microenvironments. Specifically, intracellular cholesterol is generally regulated through the SREBP-1 and SREBP-2 proteins, which, under cholesterol-depleted conditions, induce the expression of proteins responsible for cholesterol uptake and synthesis [291]. The activity of the SREBP pathway should therefore be examined as the mechanism by which SR-B1-antagonized cells upregulate HMGCR expression and vice versa with SR-B1 overexpression following statin inhibition of HMGCR. For cells unable to replenish cholesterol, cellular dysfunction could impact function through several processes, including impaired membrane synthesis and lipid-raft mediated signaling leading to the manifestation of cell stress responses [292,293].

Autophagy can manifest in response to several disruptions to cellular-signaling processes. This review describes the apparent induction of ERS and the UPR in response to SR-B1 antagonism as measured through increased expression of BiP and IRE1α. ERS is generally thought to be the result of a disruption in protein homeostasis [294] but has also been demonstrated in response to disrupted lipid homeostasis [295]. Further, ERS is known to induce autophagy, which could mechanistically explain the SR-B1-antagonized phenotype [295]. There are several proposed mechanisms of ERS-mediated autophagy that could be investigated to assess the connection, including eukaryotic initiation factor 2 (eIF2α)-autophagy-related protein 12 (Atg12) expression, IRE1α-TNF receptor-associated factor 2 (TRAF2)-c-Jun N-terminal kinase (JNK) expression, and measuring cellular Ca^2+^ and calmodulin-dependent protein kinase kinase 2 (CaMKK-β).

Although generally considered to be cholesterol-poor, increased humoral cholesterol has been demonstrated in mitochondria and may indicate a role in sustained oncogenic signaling [296]. It has been postulated that this increased cholesterol plays a role in facilitating hypoxia-inducible factor 1-alpha (HIF1α) activity and mitochondrial function in the hypoxic environment generally observed in cancer [297]. The loss of this cholesterol could result in decreased HIF1α signaling and increased hypoxic stress, leading to the observed induction of autophagy with SR-B1 antagonism [297,298]. To assess this, general measurements of mitochondrial function could be taken including ATP production and reactive oxygen species concentration or more specific assessment of HIF1α signaling.

Cholesterol constitutes a critical component of lipid rafts, which are membrane domains essential for several proliferative signaling pathways [296]. These include receptor tyrosine kinases, insulin-like growth factor 1 receptor (IGFR), and epidermal growth factor receptor (EGFR) lipid raft signaling and the small GTPase Ras family with established roles in PCa proliferation [299,300,301,302]. Both IGFR and EGFR are known to induce cancer proliferation through PI3K/AKT and Ras/MAPK signaling pathways, the disruption of which can lead to the induction of autophagy [303,304]. PI3K functions to phosphorylate phosphatidylinositol 4,5-bisphosphate (PI[4,5]P2) to PI[3–5]P3, leading to a conformational change inducing AKT binding and activation that leads to a proliferative phenotype through many different branches including increased growth through mTOR activation, increased nutrient metabolism through the inhibition of glycogen synthase kinase 3 (GSK3), and decreased apoptotic signaling through the activation of nuclear factor kappa-light-chain-enhancer of activated B cells (NF-κB) [305]. Ras is a small GTPase, which, in its active state, recruits and activates a kinase signaling cascade through RAF and MAPK kinases, leading to the phosphorylation of numerous targets including pro-proliferation transcription factors Fos, Jun, and Myc [306,307]. These pathways are considered some of the most critical oncogenic pathways, the disruption of which through impaired lipid raft formation could lead to reduced proliferative signaling and the induction of autophagy. Not to be overlooked, SR-B1 is also capable of inducing PI3K/AKT and Ras/MAPK signaling via Src independent of its effects on cellular cholesterol [211,212]. The examination of the activity of these pathways would provide insight into their role in the induction of autophagy in SR-B1-antagonized cells. However, determining whether the altered activity of these pathways is a result of cholesterol-mediated or independent effects would be necessary. Both fluorescent-probe and detergent-resistance assessment could be used to directly assess membrane formation in SR-B1-antagonized cells [308,309]. In general, understanding alterations in cellular signaling processes that drive the observed autophagic phenotype in response to SR-B1 antagonism provides not only valuable mechanistic insight into the role of SR-B1 in PCa but may also guide the design of novel small molecule SR-B1 inhibitors while providing rational candidates for synthetic lethal co-targeting.

Synthetic lethal co-targeting, which is already in development, can be approached by either high-throughput identification or rational design as a method to increase the cytotoxicity of individual cholesterol-metabolism-targeted agents [310,311]. In theory, high-throughput approaches would employ genome-wide knockout libraries in concert with SR-B1 antagonism to identify candidates resulting in robust cytotoxicity. Examples of the potential of rational co-targeting of SR-B1 with either HMGCR or autophagy inhibitors have previously been demonstrated by us [250]. Chloroquine and hydroxychloroquine, which are clinically used prophylactically against malaria, together inhibit the lysosomal degradation of cellular components of autophagic vacuoles and are commonly employed as an in vitro autophagy inhibitor [312]. Clinically, several trials are currently being undertaken combining chloroquine or hydroxychloroquine with standard therapy in an effort to prevent therapy-induced autophagic resistance in several cancer types [313]. Of those trials that have reported their findings, largely in limited numbers of glioblastoma multiforme patients, minimal to modest improvements in outcomes have been reported [314,315,316,317,318,319]. Although the clinical potential of chloroquine or hydroxychloroquine appears limited, their use for pre-clinical proof of principal experiments in bladder cancer and glioblastoma cells, respectively, are still valuable [320,321]. Furthermore, more potent autophagy inhibitors have been developed, which may demonstrate superior co-targeting than traditional agents [281,322].

SR-B1-antagonized cells show an enhanced upregulation of CLU, a stress-activated chaperone that regulates protein homeostasis by preventing protein aggregation, enhancing autophagosome biogenesis, inhibiting apoptosis, and promoting the survival of PCa [281,323]. To determine if CLU expression affects SR-B1-antagonism-induced cell stress, SR-B1-antagonized cells could be targeted with either CLU-targeted siRNA or OGX-011 antisense oligonucleotide. Evaluating these co-targeting approaches or those identified through high-throughput approaches in a similar manner to the experiments described in this review would likely provide therapeutic options for inducing a more robust cytotoxic response in SR-B1-antagonized cells. The research proposed here could further the understanding of the role and potential of targeting cholesterol metabolism in PCa, through expanding both the knowledge of clinical expression and the impact and effects of targeting cholesterol metabolism pre-clinically [281].

## Figures and Tables

**Table 1:** Therapies that have been developed due to their effect on cholesterol metabolism by therapeutic target.

Target	Mechanism of Action	Drug Class	Examples	Reference
HMG-CoA Reductase	Blocks hepatic cholesterol synthesis by inhibiting HMG-CoA reductase from converting HMG-CoA into mevalonic acid, a cholesterol precursor. This results in an increase in the number of LDL receptors, which leads to a reduction in LDL levels.	Statins (HMG-CoA reductase inhibitors)	Atorvastatin, rosuvastatin	[100,101]
PCSK9	A monoclonal antibody that binds specifically to PCSK9, preventing it from degrading LDL receptors, ultimately decreasing LDL levels in blood by increasing the number of LDL receptors available to clear LDL from the body.	PCSK9 inhibitors	Alirocumab, evolocumab	[102,103]
CYP17A1	An androgen synthesis inhibitor that works by selectively inhibiting CYP17A1, which is an enzyme required for androgen synthesis in prostatic tumor tissue, among other tissues.	CYP17A1 inhibitor (androgen biosynthesis inhibitor)	Abiraterone acetate	[104]

**Table 2:** Summary of studies on statin use and PCa from 2004 to 2020.

Reference	Objective	Study Group	Conclusion
Graaf et al. (2004) [129]	To compare the risk of cancer incidence between users of statins and users of other cardiovascular medication	PHARMO database, containing drug-dispensing records from community pharmacies and linked hospital discharge records for residents of eight Dutch cities Study base included all patients with one or more prescriptions for cardiovascular drugs in the period between 1 January 1985 and 31 December 1998	3129 patients were identified and matched to 16,976 controls Statin use was associated with a risk reduction in cancer of 20% Data suggest that statins are protective when used longer than 4 years Observational study suggests that statins may have a protective effect against cancer
Friis et al. (2005) [130]	To study if Hydroxymethylglutaryl-CoA reductase inhibitors (statins) are linked with potential chemopreventive effects	Population-based cohort study Data from the Prescription Database of North Jutland County and the Danish Cancer Registry for the 1989–2002 period	Individuals prescribed statins experienced a slightly reduced cancer incidence compared to population controls of nonusers and users of other lipid-lowering drugs Larger and longer-term studies are needed to determine the potentially protective effect of statin use on cancer development
Shannon et al. (2005) [131]	Case-control study to elucidate the association between statin use and PCa risk	PCa cases (*n* = 100), recruited upon referral for prostate biopsy, and frequency age-matched, prostate-specific antigen-normal clinic controls (*n* = 202) were recruited from the Portland, Oregon, Veterans Affairs Medical Center Information on any use of statins from May 1997 through August 2004 was obtained from an electronic pharmacy database	Statin use associated with a significant reduction in PCa risk In analyses stratified by Gleason score, the inverse association with statin use was maintained only among men with Gleason scores of > or =7 Results suggest that statins may reduce the risk of total PCa and, specifically, more aggressive PCa
Singal et al. (2005) [132]	To study the association of PCa and statins	VISN 16 data warehouse, which contains clinical and demographic information about all veterans (>1.4 million patients) cared for at the 10 VA Medical Centers in 4 states comprising the South-Central VA Health Care Network in the mid-south region of the US, was queried from October 1998 to June 2004	Statins are protective against the development of PCa after controlling for age, body mass index, smoking, diabetes, and race
Platz et al. (2006) [133]	To investigate the association of statin use with total and advanced PCa; the latter being the most important endpoint to prevent	Analyzed data from an ongoing prospective cohort study of 34,989 US male health professionals who were cancer-free in 1990 and were followed to 2002	In this cohort of male health professionals, use of statin drugs was not associated with a risk of PCa overall but was associated with a reduced risk of advanced (especially metastatic or fatal) PCa
Flick et al. (2007) [168]	To examine the association between statin use and the risk of PCa	69,047 eligible participants from the California Men’s Health Study, a prospective cohort of Northern and Southern California Kaiser Permanente (KP) members, aged 45 to 69 years, initiated in 2002 PCa identified by linkage to the KP California Cancer Registries Statin exposure, estimated from automated KP outpatient pharmacy records (available since 1991 in Southern California and since 1994 in Northern California), treated as time-varying and defined as the cumulative days dispensed of any statin from the first dispensing until a PCa diagnosis, radical prostatectomy, termination of membership, or end of study (31 December 2004)	Findings suggest long-term statin use might be associated with a reduced risk of PCa Association may only be among regular nonsteroidal anti-inflammatory drug users
Murtola et al. (2007) [134]	To evaluate the association between serum cholesterol-lowering medication use and PCa risk at the population level	All newly diagnosed PCa cases in Finland during 1995 to 2002 and matched controls identified from Finnish Cancer Registry and the Population Register Center, respectively Detailed information on cholesterol-lowering drug purchases during the study period obtained from the prescription database of the Social Insurance Institution of Finland	Large population-based study showed: Risk of advanced cancer decreased among statin users No evidence for reduced overall PCa risk among users of cholesterol-lowering drugs
Jacobs et al. (2007) [135]	To examine the association between the use of cholesterol-lowering drugs and PCa incidence by disease stage and grade	55,454 men in the Cancer Prevention Study II Nutrition Cohort	Results provide some support for the hypothesis that long-term statin use is associated with reduced risk of advanced PCa
Bonovas et al. (2008) [136]	To examine statin use in relation to both total PCa and the more clinically important advanced PCa epidemiologic studies published on the subject in peer-reviewed literature	19 studies included in review: 6 randomized clinical trials 13 observational studies (six cohort studies and seven case control studies, *n* = 840,000 men)	No evidence of an association between statin use and total PCa There was a protective association between statin use and advanced PCa
Breau et al. (2010) [137]	To evaluate the effect of statin medication use on the risk of PCa	Data from a longitudinal, population-based cohort of 2447 men between 40 and 79 years old followed from 1990 to 2007 were examined Information on statin use was self-reported and obtained by biennial questionnaires Randomly selected subset of men completed biennial urological examinations that included serum prostate-specific antigen measurements Information on prostate biopsy and PCa obtained through review of community medical records	Statin use is associated with a decreased risk of PCa diagnosis Association may be explained by decreased detection or cancer prevention
Murtola et al. (2010) [138]	To compare the relative risk of advanced PCa between current users and non-users of statins or other cholesterol-lowering medications	Study cohort comprised of 23,320 men participating in screening arm of the Finnish PCa screening trial during 1996–2004 Information on medication use obtained from a comprehensive national prescription database Compared relative risk between current users and non-users of statins or other cholesterol-lowering medications in a population undergoing systematical PCa screening	Overall incidence of PCa was lowered among statin users Cholesterol-lowering with statins seems beneficial for PCa prevention
Farwell et al. (2011) [139]	To determine if statins can be used as a prevention strategy for high-grade PCas	55,875 men taking either a statin or antihypertensive medication identified from electronic and administrative files from the Veterans Affairs New England Healthcare System	Statin use is associated with statistically significantly reduced risk for total and high-grade PCa Increased levels of serum cholesterol are associated with higher risk for total and high-grade PCa
Tan et al. (2011) [140]	To determine the association between statin use and PCa men who underwent prostate biopsy	A retrospective cohort study of men who underwent prostate biopsy from 2000 to 2007 at Cleveland Clinic Statin use determined using outpatient pharmacy record Clinical and pathological outcomes were obtained	Statin use was associated with a decreased risk of PCa, less frequent high-grade PCa, and a lower volume of PCa Statin use has a protective effect against PCa
Bansal et al. (2012) [141]	To examine the association between statin use and risk of PCa by conducting a detailed meta-analysis of all observational studies published regarding this subject	Meta-analysis of observational studies evaluating the association between statin use and risk of PCa via a literature search (PubMed database) undertaken through February 2012	Statins reduce the risk of both total PCa and clinically important advanced PCa
Marcella et al. (2012) [150]	Population-based case-control investigation to specifically examine the association between statin use and PCa mortality	Matched case-control study of residents of New Jersey aged 55–79 who died from PCa between 1997–2000 Individually matched population-based controls by five-year age-group and race Medication data were obtained identically for cases and controls from blinded medical chart review	Statin use is associated with substantial protection against PCa death, adding to the epidemiologic evidence for an inhibitory effect on PCa
Nielsen et al. (2012) [151]	To test the hypothesis that statin use begun before a cancer diagnosis is associated with reduced cancer-related mortality	Mortality among patients from the entire Danish population who had received a diagnosis of cancer between 1995 and 2007 assessed, with follow-up until 31 December 2009 Patients 40 years of age or older; 18,721 had used statins regularly before the cancer diagnosis, and 277,204 had never used statins	Statin use in patients with cancer is associated with reduced cancer-related mortality.
Geybels et al. (2013) [148]	To investigate the associations between statin use begun before PCa (PCa) diagnosis and PCa recurrence/progression and PCa-specific mortality (PCSM) in a prospective, population-based cohort study	Analysis of 1001 PCa patients diagnosed in 2002–2005 in King County, Washington Statin use was assessed at the time of diagnosis using a detailed in-person interview PCa recurrence/progression events and cause-specific survival ascertained from a follow-up survey and SEER registry Associations between statin use begun before PCa (PCa) diagnosis and PCa recurrence/progression, and PCa-specific mortality (PCSM) was investigated	Statin use begun before PCa diagnosis was unrelated to PCa recurrence/progression but was associated with a decrease in risk of PCSM
Park et al. (2013) [147]	Meta-analysis to evaluate associations between statins and recurrence-free survival (RFS) following treatment of localized PCa, with attention to potential benefits among patients treated primarily with radiotherapy (RT) versus radical prostatectomy	Seventeen studies examining the effect of statins on men who received definitive treatment of localized PCa using a systematic search of the PubMed and EMBASE databases through August 2012 Meta-analysis that evaluated the associations between statins and recurrence-free survival (RFS) following treatment of localized PCa, with attention to potential benefits among patients treated primarily with radiotherapy (RT) versus radical prostatectomy	Meta-analysis suggests a potentially beneficial effect of statins on PCa patients treated with RT but not among radical prostatectomy patients
Yu et al. (2014) [153]	To determine whether the use of statins after PCa diagnosis is associated with a decreased risk of cancer-related mortality and all-cause mortality and to assess whether this association is modified by prediagnostic use of statins	Cohort of 11,772 men newly diagnosed with non-metastatic PCa between 1 April 1998, and 31 December 2009, followed until 1 October 2012 Cohort identified using a large population-based electronic database from the United Kingdom. To determine whether the use of statins after PCa diagnosis associated with a decreased risk of cancer-related mortality and all-cause mortality and to assess whether this association is modified by prediagnostic use of statins	Use of statins after diagnosis associated with decreased risk in PCa mortality Effect was stronger in patients who also used statins before diagnosis
Grytli et al. (2014) [152]	To assess the association between β-blockers and PCa-specific mortality in patients with high-risk or metastatic disease and to address potential confounding from the use of statins or acetylsalicylic acid (ASA)	Cohort of 3561 PCa patients with high-risk or metastatic disease Clinical information from all men reported to the Cancer Registry of Norway with a PCa diagnosis between 2004 and 2009 (*n* = 24,571) Information on filled prescriptions between 2004 and 2011 from the Norwegian Prescription Database	β-Blocker use was associated with reduced PCa-specific mortality in patients with high-risk or metastatic disease at the time of diagnosis
Jespersen et al. (2014) [142]	To examine the association between statin use and risk of PCa in a Denmark-based case-control study	42,480 patients diagnosed with incident PCa during 1997–2010 from a national cancer registry Five age-matched population controls selected for each case using risk-set sampling Statin use from 1996 to the index date was obtained from the National Prescription Registry	Statin use was associated with a risk reduction overall (6%) and, specifically, with advanced PCa (10%)
Lustman et al. (2014) [143]	To look for evidence for long-term statin use as an effective chemoprevention for PCa	Population-based cohort study examined the association between statin use and risk of PCa using the Database of Clalit Health Services A total of 66,741 eligible participants were identified at 1 January 2001 and followed through to 31 December 2009	Prolonged statin use was associated with a reduced risk of PCa; however, this was not true for all types of statin
Harshman et al. (2015) [149]	To evaluate whether statin use prolongs TTP during ADT for hormone-sensitive PCa	Statin use retrospectively analyzed in 926 patients who received androgen deprivation therapy (ADT) for biochemical or metastatic recurrence or de novo metastatic PCa between January 1996 and November 2013 Evaluated whether statin use prolongs time to progression (TTP) during ADT for hormone-sensitive PCa	Statin use at the time of ADT initiation was associated with a significantly longer TTP during ADT even after adjustment for known prognostic factors
Larsen et al. (2017) [154]	To examine whether postdiagnosis statin use was associated with reduced cancer-specific mortality or all-cause mortality among patients with PCa in Denmark	All patients identified through nationwide Danish registries with incident prostate adenocarcinoma from 1998 to 2011 and retrieved data on tumor and patient characteristics, drug use, and primary treatment Post-diagnosis use was defined as two or more prescriptions of statins as a time-varying covariate with a 1-year lag	Post-diagnosis statin use was associated with reduced mortality from PCa; however, it remains to be established whether this association is causal
Van Rompay et al. (2019) [144]	To test the hypothesis that cholesterol-lowering drugs affect PC incidence and severity	A retrospective cohort study conducted by abstracting prescription and health service records for 249,986 Saskatchewan men aged ≥40 years between 1 January 1990 and 31 December 2014	Analyses provide comprehensive findings that statins may reduce risk of metastatic PC and PC mortality Study also demonstrates NSLLM have similar effects, supporting a cholesterol-based mechanism
Allot et al. (2020) [145]	To examine statins and the risk of lethal PCa in the Health Professionals Follow-up Study (HPFS), test associations with molecular subtypes, and integrate gene expression profiling to identify putative mechanisms	Prospectively examined statins and lethal PCa risk in Health Professionals Follow-up Study (HPFS) Tested associations with molecular subtypes and integrated gene expression profiling to identify putative mechanisms Study included 44,126 men cancer-free in 1990, followed for PCa incidence through 2014, with statin use recorded on biennial questionnaires	Molecular tumor classification identified PTEN and inflammation/immune activation as potential mechanisms linking statins with lower lethal PCa risk Findings supported a potential causal association and could inform selection of relevant biomarkers for statin clinical trials
**Studies Demonstrating No Link Between Incidence of PCa with Use of Statins**			
**Reference**	**Objective**	**Study Group**	**Conclusion**
Boudreau et al. (2008) [166]	To evaluate the relation between statin use and PCa risk	A retrospective cohort study during 1 January 1990 to 31 August 2005 among men aged 45–79 years receiving care within Group Health, an integrated healthcare delivery system PCa cases were identified through the Surveillance, Epidemiology, and End Results cancer registry Information on statin use and covariates was obtained from the health plan database	Study did not support any associated between statin use and PCa but concluded that a reduced risk could not be ruled out
Friedman et al. (2008) [155]	To determine the risk of cancer in statin users	Risk of cancer in up to 9.4 years after first recorded receipt of statins evaluated in subscribers of an integrated health care program in northern California Statin use and cancer development ascertained from the program’s pharmacy records and cancer registry from August 1994 to December 2003	Study provided no strong evidence of either causation or prevention of cancer by statins
Kuoppala et al. (2008) [156]	To review the overall evidence on the association between statin therapy and cancer	42 studies included in the analyses: ○ 17 randomized controlled trials, ○ 10 cohort studies, and ○ 15 case-control studies	Evidence suggested that statins did not have short-term effects on cancer risk Evidence on the potentially protective or harmful effects was inconclusive
Smeeth et al. (2009) [157]	To assess the effect of statins on a range of health outcomes	A population-based cohort from the United Kingdom Health Improvement Network database Included computerized medical records of over four and a half million patients Assessed the effect of statins on a range of health outcomes between people prescribed and not prescribed statins	Found little evidence to support wide-ranging effects of statins on health outcomes beyond their established beneficial effect on vascular disease
Coogan et al. (2010) [165]	To update the findings by cancer stage and to evaluate the joint use of statins and NSAIDs	Cases of 1367 men with PCa compared to controls cases of 2007 men with diagnoses unrelated to statin or NSAID use	Results do not support a protective effect of statin use, or statin and NSAID use, on the risk of advanced PCa
Haukka et al. (2010) [158]	To examine the association between consumption of statins and the risk of cancer, including PCa	A record-linkage study in Finland utilizing nationwide databases of reimbursed statin medication and cancer The study population included all statin users in Finland who had purchased at least one prescription between 1996 and 2005 and who had no cancer diagnosis at the date of first purchase A control population without statin usage was included	Found neither beneficial nor harmful associations between the usage of statins and cancer including PCa
Hippisley-Cox et al. (2010) [159]	To quantify the unintended effects of statins according to type, dose, and duration of use	2,004,692 patients aged 30–84 years, of whom 225,922 were new users of statins: ○ 159,790 were prescribed simvastatin; ○ 50,328 were prescribed atorvastatin; ○ 8103 were prescribed pravastatin, ○ 4497 were prescribed rosuvastatin; and ○ 3204 prescribed fluvastatin	Individual statin were not significantly associated with a risk of PCa
Fowke et al. (2011) [164]	To investigate the association between statin use and the likelihood of having a PSA or DRE test, blood PSA levels, prostate volume, and the severity of lower urinary tract symptoms	A multicenter, rapid recruitment protocol was used to collect clinical, biologic, behavioral, and body measurement data from 2148 men 40 years or older scheduled for diagnostic prostate biopsy Medication use and other data were ascertained by research survey, clinical interview, and chart review	Results suggested that selective referral for biopsy associated with statin use is an essential element to address in further understanding the potential for statins to prevent PCa
Jacobs et al. (2011) [160]	To examine the association between the long-term use of cholesterol-lowering drugs, predominantly statins, and the incidence of ten common cancers including PCa, as well as overall cancer incidence	133,255 participants (60,059 men and 73,196 women) in the Cancer Prevention Study II Nutrition Cohort during the period from 1997 to 2007	Results suggested that long-term use of statins unlikely to substantially increase or decrease overall cancer risk
Chan et al. (2012) [161]	To examine the evidence of an association between statins and PCa risk	Statin use in a prospective cohort of 5069 elderly U.S. men and the risk of incident total, low/high stage, and low/high grade PCa diagnoses between 2000 and 2008	Study of elderly U.S. men; a null association was observed between statin use and risk of PCa
Freedland et al. (2013) [162]	To examine the association between statins and cancer and high-grade cancer in REDUCE, where biopsies were largely PSA-independent	Analysis of REDUCE—a prospective multinational randomized controlled trial of dutasteride vs. placebo for 4 years among men aged 50–75 years with a PSA of 2.5–10.0 ng mL^−1^ and a negative biopsy at baseline; it included PSA-independent biopsies mandated at 2- and 4-years Analyses were limited to men who underwent at least one biopsy while under study Association between baseline statin use and risk of overall, high-grade (Gleason ≥ 7), or low-grade (Gleason ≤ 6) PC vs. no cancer was examined using multinomial logistic regression	Among men with a negative baseline biopsy and follow-up biopsies largely independent of PSA, statins were not associated with cancer or high-grade cancer
Plat et al. (2014) [163]	To investigate whether statin drug use influences the risk of screen detected PCa in a setting in which men had a low baseline serum PSA concentration and were screened annually	Cohort study of 9457 men 55 years old or older at randomization to the placebo arm of the PCa prevention trial (PCPT) Men reported new use of medications quarterly	Prospective results do not support the hypothesis that statin drugs protect against PCa in the setting of a regular PCa screening
Fulcher et al. (2015) [107]	To examine the accuracy of popular risk calculators amongst primary prevention, control arm patients of statin trials in the Cholesterol Treatment Trialists’ Collaboration (CTTC) database	22 trials of statin therapy versus control and 5 trials of more-intensive versus less-intensive statin therapy	No adverse effect on rates of cancer incidence or non-cardiovascular mortality were noted
Kantor et al. (2015) [146]	To evaluate the association between statin use and PCa risk in the Southern Community Cohort Study (SCCS)	Study participants were 32,091 men aged 40–79 at baseline, 67% of whom were non-Hispanic black Between study enrolment (2002–2009) and 31 December 2010, 570 PCa cases were diagnosed, including 324 low-grade cancers (Gleason score <7 or Gleason pattern 3 + 4) and 107 high-grade cancers (Gleason score >7 or Gleason pattern 4 + 3)	Results suggest no strong association between statin use and PCa risk overall If a modest protective effect does exist, it does not vary by race/ethnicity and may be restricted to high-grade tumors, although the power to detect differences by subgroup was limited
**Study Demonstrating Increased Risk of PCa with Use of Statins**			
**Reference**	**Objective**	**Study Group**	**Conclusion**
Chang et al. (2011) [167]	To investigate whether the use of statins was associated with PCa risk	Population-based case-control study in Taiwan Data retrospectively collected from Taiwan National Health Insurance Research Database Cases consisted of all patients who were aged 50 years and older and had a first-time diagnosis of PCa for the period between 2005 and 2008 Controls matched to cases by age, sex, and index date	Results from case-control study suggested that statins may increase the risk of PCa

**Table 3:** SR-B1 as a potential therapeutic target for management of CRPC.

Study	Objectives	Key Findings	Conclusions
Leon et al. (2010) [248]	To assess if cholesterol and its regulation are altered in CRPC cells using a murine PCa xenograft model	Proteins involved in cholesterol regulation (e.g., SR-B1, etc.) are altered during disease progression to increase uptake and intra-/extra-tumoral production of cholesterol Post-castration, this can provide an increased amount of precursor for intratumoral steroidogenesis. Androgens increase in CRPC leading to induction of AR expression and PSA production	During progression to CRPC, cholesterol regulation processes are altered Increased production and/or uptake of cholesterol is likely a precursor for intratumoral de novo synthesis
Twiddy et al. (2012) [249]	To silence SRB1 and assess the ability of PCa cells to maintain internal supplies of androgens without the presence of cholesterol	Silencing SR-B1 (to >85%) decreased PSA production in LNCaP and C4-2 SRB1-KD cells by 55% and 58%, respectively, compared to controls Additionally, C4-2 cells had reduced cell viability (>25%)	PSA production and cell viability were reduced in C4-2 cells following SR-B1 downregulation with or without HDL This may indicate a deficiency of cholesterol availability to the androgen synthesis pathway or a role for SR-B1 in the PC signal transduction pathway
Schörghofer et al. (2015) [90]	To study SR-B1 expression in clinical PCa samples	Identification of an association of PCa and SR-B1 mRNA and protein expression. High Gleason-grade PCa samples demonstrated significantly higher SR-B1 expression as well as higher SR-B1 mRNA expression in metastatic versus non-metastatic PCa SR-B1 staining intensity was higher in PCa biopsies (53.6%) compared to non-cancerous samples (0%)	SR-B1 was identified as an indicator of human PCa formation, meaning that increased levels may be involved with the generation of a castration-resistant phenotype Therapeutic inhibition of SR-B1 may inhibit PCa progression. There was a positive correlation of SR-B1 expression and that of androgen-producing enzymes and mTOR activation
Patel et al. (2018) [251]	To demonstrate that a SPRY2 deficiency leads to treatment resistance and an androgen self-sufficient CRPC To identify important processes that influence treatment response in CRPC	Cholesterol transport blockade using an SRB1 antagonist (ITX5061) was found to safely decrease treatment resistance in SPRY-2 deficient CRPC	ITX5061 and statins when used at specific nodes of PCa disease progression may be used to sensitize tumors to ADT in the treatment of CRPC IL6 is a systemic prognostic marker, and SR-B1 is an actionable target in this disease state
Gordon et al. (2019) [250]	To demonstrate that SR-B1 expression may contribute to malignant transformation by increasing levels of available cholesterol To identify SR-B1 expression in the progression from normal to cancerous prostatic tissue and to CRPC To see the effect of SR-B1 antagonism in PCa cell lines	Increased SR-B1 protein and transcript expression in PCa relative to normal prostate epithelium, and high SR-B1 expression in CRPC metastasis Using the androgen-responsive CRPC cell model (C4-2) that antagonism of SR-B1 suppresses cholesterol uptake, de novo steroidogenesis, AR activity, growth and viability and induces endoplasmic reticulum stress and autophagy AR pathway activation is insufficient to overcome cytotoxic stress caused by decreased cholesterol availability	SR-B1 antagonism was shown to impact CRPC growth SR-B1 is an important contributing factor in the sustained proliferation of malignant prostatic disease and thus highlights the potential for the development of a novel SR-B1 inhibitor designed for in vivo use The ability of SR-B1 antagonism to arrest growth independent of AR activity, while also reducing AR activity in steroid-responsive PCa, provides a promising therapeutic prospect across the CRPC spectrum
Traughber et al. (2020) [252]	To examine the effect of HDL on PCa cell growth, proliferation, and tumor progression	SR-B1 knockout in PCa tumors decreased HDL-associated PCa cell proliferation and disease progression by reducing HDL uptake	Cholesterol metabolism may have an important role in the treatment of PCa As SR-B1 and HDL uptake promoted PCa progression, this suggests that HDL uptake inhibition may be a viable target for decreasing disease burden

## References

[1] Sung H., Ferlay J., Siegel R.L., Laversanne M., Soerjomataram I., Jemal A., Bray F. (2021). Global Cancer Statistics 2020: GLOBOCAN Estimates of Incidence and Mortality Worldwide for 36 Cancers in 185 Countries. CA Cancer J. Clin..

[2] Litwin M.S., Tan H.J. (2017). The Diagnosis and Treatment of Prostate Cancer: A Review. JAMA.

[3] Teo Y.M., Rathkopf D.E., Kantoff P. (2019). Treatment of Advanced Prostate Cancer. Annu. Rev. Med..

[4] Mansinho A., Macedo D., Fernandes I., Costa L. (2019). Castration-Resistant Prostate Cancer: Mechanisms, Targets and Treatment. Prostate Cancer.

[5] Crowley F., Sterpi M., Buckley C., Margetich L., Handa S., Dovey Z. (2021). A Review of the Pathophysiological Mechanisms Underlying Castration-resistant Prostate Cancer. Res. Rep. Urol..

[6] Vellky J.E., Ricke W.A. (2020). Development and prevalence of castration-resistant prostate cancer subtypes. Neoplasia.

[7] Sumanasuriya S., De Bono J. (2018). Treatment of Advanced Prostate Cancer—A Review of Current Therapies and Future Promise. Cold Spring Harb. Perspect. Med..

[8] Feng Q., He B. (2019). Androgen Receptor Signaling in the Development of Castration-Resistant Prostate Cancer. Front. Oncol..

[9] Montgomery R.B., Mostaghel E.A., Vessella R., Hess D.L., Kalhorn T.F., Higano C.S., True L.D., Nelson P.S. (2008). Maintenance of Intratumoral Androgens in Metastatic Prostate Cancer: A Mechanism for Castration-Resistant Tumor Growth. Cancer Res..

[10] Locke J.A., Guns E.S., Lubik A.A., Adomat H.H., Hendy S.C., Wood C.A., Ettinger S.L., Gleave M.E., Nelson C.C. (2008). Androgen Levels Increase by Intratumoral de novo Steroidogenesis during Progression of Castration-Resistant Prostate Cancer. Cancer Res..

[11] Fontana F., Limonta P. (2021). Dissecting the Hormonal Signaling Landscape in Castration-Resistant Prostate Cancer. Cells.

[12] Jung M.E., Ouk S., Yoo D., Sawyers C.L., Chen C., Tran C., Wongvipat J. (2010). Structure-Activity Relationship for Thiohydantoin Androgen Receptor Antagonists for Castration-Resistant Prostate Cancer (CRPC). J. Med. Chem..

[13] Narayanan R. (2020). Therapeutic targeting of the androgen receptor (AR) and AR variants in prostate cancer. Asian J. Urol..

[14] Potter G.A., Barrie S.E., Jarman M., Rowlands M.G. (1995). Novel Steroidal Inhibitors of Human Cytochrome P450(17-Alpha) (17-Alpha-Hydroxylase-C-17,C-20-Lyase)—Potential Agents for the Treatment of Prostatic-Cancer. J. Med. Chem..

[15] Duarte C., Jimeno A., Kessler E. (2019). Abiraterone acetate to treat metastatic castration-resistant prostate cancer in combination with prednisone. Drugs Today.

[16] Mitsiades N., Kaochar S. (2021). Androgen receptor signaling inhibitors: Post-chemotherapy, pre-chemotherapy and now in castration-sensitive prostate cancer. Endocr. Relat. Cancer.

[17] Velho P.I., Bastos D.A., Antonarakis E.S. (2021). New approaches to targeting the androgen receptor pathway in prostate cancer. Clin. Adv. Hematol. Oncol..

[18] Small E.J. (2017). Redefining Hormonal Therapy for Advanced Prostate Cancer: Results from the LATITUDE and STAMPEDE Studies. Cancer Cell.

[19] Mori K., Miura N., Mostafaei H., Quhal F., Motlagh R.S., Pradere B., Kimura S., Kimura T., Egawa S., Briganti A. (2020). Sequential therapy of abiraterone and enzalutamide in castration-resistant prostate cancer: A systematic review and meta-analysis. Prostate Cancer Prostatic Dis..

[20] Fizazi K., Kramer G., Eymard J.-C., Sternberg C.N., de Bono J., Castellano D., Tombal B., Wülfing C., Liontos M., Carles J. (2020). Quality of life in patients with metastatic prostate cancer following treatment with cabazitaxel versus abiraterone or enzalutamide (CARD): An analysis of a randomised, multicentre, open-label, phase 4 study. Lancet Oncol..

[21] Presicce F., Giacinti S., Bassanelli M., Tubaro A. (2018). Castration-resistance prostate cancer: What is in the pipeline?. Minerva Urol. Nefrol..

[22] Watson P.A., Arora V.K., Sawyers C.L. (2015). Emerging mechanisms of resistance to androgen receptor inhibitors in prostate cancer. Nat. Rev. Cancer.

[23] Snow O., Lallous N., Singh K., Lack N., Rennie P., Cherkasov A. (2019). Androgen receptor plasticity and its implications for prostate cancer therapy. Cancer Treat. Rev..

[24] Zhang T., Karsh L.I., Nissenblatt M.J., Canfield S.E. (2020). Androgen Receptor Splice Variant, AR-V7, as a Biomarker of Resistance to Androgen Axis-Targeted Therapies in Advanced Prostate Cancer. Clin. Genitourin. Cancer.

[25] Zhu Y., Dalrymple S.L., Coleman I., Zheng S.L., Xu J., Hooper J.E., Antonarakis E.S., De Marzo A.M., Meeker A.K., Nelson P.S. (2020). Role of androgen receptor splice variant-7 (AR-V7) in prostate cancer resistance to 2nd-generation androgen receptor signaling inhibitors. Oncogene.

[26] Antonarakis E.S., Lu C., Wang H., Luber B., Nakazawa M., Roeser J.C., Chen Y., Mohammad T., Chen Y., Fedor H.L. (2014). AR-V7 and Resistance to Enzalutamide and Abiraterone in Prostate Cancer. N. Engl. J. Med..

[27] Sharp A., Coleman I., Yuan W., Sprenger C., Dolling D., Rodrigues D.N., Russo J.W., Figueiredo I., Bertan C., Seed G. (2019). Androgen receptor splice variant-7 expression emerges with castration resistance in prostate cancer. J. Clin. Investig..

[28] Schmidt K.T., Huitema A.D.R., Chau C.H., Figg W.D. (2021). Resistance to second-generation androgen receptor antagonists in prostate cancer. Nat. Rev. Urol..

[29] Labrecque M., Coleman I.M., Brown L.G., True L.D., Kollath L., Lakely B., Nguyen H.M., Yang Y.C., Gil Da Costa R.M., Kaipainen A. (2019). Molecular profiling stratifies diverse phenotypes of treatment-refractory metastatic castration-resistant prostate cancer. J. Clin. Investig..

[30] Beltran H., Prandi D., Mosquera J.M., Benelli M., Puca L., Cyrta J., Marotz C., Giannopoulou E., Chakravarthi B.V., Varambally S. (2016). Divergent clonal evolution of castration-resistant neuroendocrine prostate cancer. Nat. Med..

[31] Obinata D., Lawrence M.G., Takayama K., Choo N., Risbridger G.P., Takahashi S., Inoue S. (2020). Recent Discoveries in the Androgen Receptor Pathway in Castration-Resistant Prostate Cancer. Front. Oncol..

[32] Moll J.M., Kumagai J., van Royen M., Teubel W.J., Van Soest R.J., French P.J., Homma Y., Jenster G., De Wit R., Van Weerden W.M. (2019). A bypass mechanism of abiraterone-resistant prostate cancer: Accumulating CYP17A1 substrates activate androgen receptor signaling. Prostate.

[33] Taplin M.-E., RajeshKumar B., Halabi S., Werner C.P., Woda B.A., Picus J., Stadler W., Hayes D.F., Kantoff P.W., Vogelzang N.J. (2003). Androgen Receptor Mutations in Androgen-Independent Prostate Cancer: Cancer and Leukemia Group B Study 9663. J. Clin. Oncol..

[34] Nacusi L.P., Tindall D.J. (2009). Androgen receptor abnormalities in castration-recurrent prostate cancer. Expert Rev. Endocrinol. Metab..

[35] Cai C., Chen S., Ng P., Bubley G.J., Nelson P.S., Mostaghel E.A., Marck B., Matsumoto A.M., Simon N., Wang H. (2011). Intratumoral de Novo Steroid Synthesis Activates Androgen Receptor in Castration-Resistant Prostate Cancer and Is Upregulated by Treatment with CYP17A1 Inhibitors. Cancer Res..

[36] Buchanan G., Irvine R.A., Coetzee G.A., Tilley W. (2001). Contribution of the Androgen Receptor to Prostate Cancer Predisposition and Progression. Cancer Metastasis Rev..

[37] Attard G., Reid A.H.M., Auchus R.J., Hughes B.A., Cassidy A.M., Thompson E., Oommen N.B., Folkerd E., Dowsett M., Arlt W. (2012). Clinical and Biochemical Consequences of CYP17A1 Inhibition with Abiraterone Given with and without Exogenous Glucocorticoids in Castrate Men with Advanced Prostate Cancer. J. Clin. Endocrinol. Metab..

[38] Vis A.N., Schröder F.H. (2009). Key targets of hormonal treatment of prostate cancer. Part 1: The androgen receptor and steroidogenic pathways. BJU Int..

[39] Vis A.N., Schröder F.H. (2009). Key targets of hormonal treatment of prostate cancer. Part 2: The androgen receptor and 5α-reductase. BJU Int..

[40] Deb S., Chin M.Y., Pham S., Adomat H., Hurtado-Coll A., Gleave M.E., Guns E.S.T. (2021). Steroidogenesis in Peripheral and Transition Zones of Human Prostate Cancer Tissue. Int. J. Mol. Sci..

[41] Cortes V.A., Busso D., Maiz A., Arteaga A., Nervi F., Rigotti A. (2014). Physiological and pathological implications of cholesterol. Front. Biosci..

[42] Iso H., Ikeda A., Inoue M., Sato S., Tsugane S., JPHC Study Group (2009). Serum cholesterol levels in relation to the incidence of cancer: The JPHC study cohorts. Int. J. Cancer.

[43] Pelton K., Freeman M.R., Solomon K.R. (2012). Cholesterol and prostate cancer. Curr. Opin. Pharmacol..

[44] Saranyutanon S., Deshmukh S.K., Dasgupta S., Pai S., Singh S., Singh A.P. (2020). Cellular and Molecular Progression of Prostate Cancer: Models for Basic and Preclinical Research. Cancers.

[45] Bott S.R.J., Ng K.L. (2021). Prostate Cancer.

[46] Pisano C., Tucci M., Di Stefano R.F., Turco F., Scagliotti G.V., Di Maio M., Buttigliero C. (2021). Interactions between androgen receptor signaling and other molecular pathways in prostate cancer progression: Current and future clinical implications. Crit. Rev. Oncol. Hematol..

[47] Aurilio G., Cimadamore A., Mazzucchelli R., Lopez-Beltran A., Verri E., Scarpelli M., Massari F., Cheng L., Santoni M., Montironi R. (2020). Androgen Receptor Signaling Pathway in Prostate Cancer: From Genetics to Clinical Applications. Cells.

[48] Luo J., Yang H., Song B.-L. (2020). Mechanisms and regulation of cholesterol homeostasis. Nat. Rev. Mol. Cell Biol..

[49] Schade D.S., Shey L., Eaton R.P. (2020). Cholesterol Review: A Metabolically Important Molecule. Endocr. Pract..

[50] Ryu S., Howland A., Song B., Youn C., Song P.I. (2020). Scavenger Receptor Class A to E Involved in Various Cancers. Chonnam Med. J..

[51] Huff T., Boyd B., Jialal I. (2021). Physiology, Cholesterol.

[52] Ference A.B., Kastelein J.J.P., Catapano A.L. (2020). Lipids and Lipoproteins in 2020. JAMA.

[53] Feingold K.R., Grunfeld C. (2000). Introduction to Lipids and Lipoproteins.

[54] Servier medical art. http://smart.servier.com/.

[55] Gomaraschi M. (2020). Role of Lipoproteins in the Microenvironment of Hormone-Dependent Cancers. Trends Endocrinol. Metab..

[56] Charlton-Menys V., Durrington P.N. (2008). Human cholesterol metabolism and therapeutic molecules. Exp. Physiol..

[57] Parthasarathy S., Raghavamenon A., Garelnabi M.O., Santanam N. (2010). Oxidized low-density lipoprotein. Methods Mol. Biol..

[58] Zhou L., Li C., Gao L., Wang A. (2015). High-density lipoprotein synthesis and metabolism. Mol. Med. Rep..

[59] Luo D.-X., Cao D.-L., Xiong Y., Peng X.-H., Liao D.-F. (2010). A novel model of cholesterol efflux from lipid-loaded cells. Acta Pharmacol. Sin..

[60] Kotani K., Sekine Y., Ishikawa S., Ikpot I.Z., Suzuki K., Remaley A.T. (2013). High-Density Lipoprotein and Prostate Cancer: An Overview. J. Epidemiol..

[61] Zhao T., Zhu N., Shi Y., Wang Y., Zhang C., Deng C., Liao D., Qin L. (2021). Targeting HDL in tumor microenvironment: New hope for cancer therapy. J. Cell. Physiol..

[62] Liang C.-Z., Fan Y.-D., Wang J., Xu L.-F., Liu C., Huang T. (2021). Identifying the role of apolipoprotein A-I in prostate cancer. Asian J. Androl..

[63] White C.P. (1909). On the occurrence of crystals in tumours. J. Pathol. Bacteriol..

[64] Swyer G.I.M. (1942). The cholesterol content of normal and enlarged prostates. Cancer Res..

[65] Pearce M.L., Dayton S. (1971). Incidence of Cancer in Men on a Diet High in Polyunsaturated Fat. Lancet.

[66] Muldoon M.F., Manuck S.B., Matthews K.A. (1990). Lowering cholesterol concentrations and mortality: A quantitative review of primary prevention trials. BMJ.

[67] Yusuf S., Wittes J., Friedman L. (1988). Overview of results of randomized clinical trials in heart disease. II. Unstable angina, heart failure, primary prevention with aspirin, and risk factor modification. JAMA.

[68] Solomon K.R., Freeman M.R. (2011). The Complex Interplay Between Cholesterol and Prostate Malignancy. Urol. Clin. N. Am..

[69] Bravi F., Scotti L., Bosetti C., Talamini R., Negri E., Montella M., Franceschi S., La Vecchia C. (2006). Self-reported history of hypercholesterolaemia and gallstones and the risk of prostate cancer. Ann. Oncol..

[70] Platz E.A., Clinton S.K., Giovannucci E. (2008). Association between plasma cholesterol and prostate cancer in the PSA era. Int. J. Cancer.

[71] Batty G.D., Kivimaki M., Clarke R., Smith G.D., Shipley M.J. (2011). Modifiable risk factors for prostate cancer mortality in London: Forty years of follow-up in the Whitehall study. Cancer Causes Control..

[72] Yannucci J., Manola J., Garnick M.B., Bhat G., Bubley G.J. (2006). The Effect of Androgen Deprivation Therapy on Fasting Serum Lipid and Glucose Parameters. J. Urol..

[73] Mohamedali H.Z., Breunis H., Timilshina N., Alibhai S.M. (2011). Changes in blood glucose and cholesterol levels due to androgen deprivation therapy in men with non-metastatic prostate cancer. Can. Urol. Assoc. J..

[74] Han W., Gao S., Barrett D., Ahmed M., Han D., Macoska J.A., He H.H., Cai C. (2018). Reactivation of androgen receptor-regulated lipid biosynthesis drives the progression of castration-resistant prostate cancer. Oncogene.

[75] Smith M.R., Finkelstein J.S., McGovern F.J., Zietman A.L., Fallon M.A., Schoenfeld D.A., Kantoff P.W. (2002). Changes in body composition during androgen deprivation therapy for prostate cancer. J. Clin. Endocrinol. Metab..

[76] Dockery F., Bulpitt C.J., Agarwal S., Donaldson M., Rajkumar C. (2003). Testosterone suppression in men with prostate cancer leads to an increase in arterial stiffness and hyperinsulinaemia. Clin. Sci..

[77] Eri L.M., Urdal P., Bechensteen A.G. (1995). Effects of the luteinizing hormone-releasing hormone agonist leuprolide on lipoproteins, fibrinogen and plasminogen activator inhibitor in patients with benign prostatic hyperplasia. J. Urol..

[78] Torimoto K., Samma S., Kagebayashi Y., Chihara Y., Tanaka N., Hirayama A., Fujimoto K., Hirao Y. (2011). The Effects of Androgen Deprivation Therapy on Lipid Metabolism and Body Composition in Japanese Patients with Prostate Cancer. Jpn. J. Clin. Oncol..

[79] Choi S.M., Kam S.C. (2015). Metabolic effects of androgen deprivation therapy. Korean J. Urol..

[80] Thysell E., Surowiec I., Hörnberg E., Crnalic S., Widmark A., Johansson A.I., Stattin P., Bergh A., Moritz T., Antti H. (2010). Metabolomic Characterization of Human Prostate Cancer Bone Metastases Reveals Increased Levels of Cholesterol. PLoS ONE.

[81] Hirano H., Ide H., Lu Y., Inoue Y., Okada H., Horie S. (2020). Impact of Pretreatment Total Cholesterol Level Is Associated With Metastasis of Prostate Cancer. Am. J. Men’s Health.

[82] Llaverias G., Danilo C., Wang Y., Witkiewicz A.K., Daumer K., Lisanti M.P., Frank P. (2010). A Western-Type Diet Accelerates Tumor Progression in an Autochthonous Mouse Model of Prostate Cancer. Am. J. Pathol..

[83] Zhuang L., Kim J., Adam R.M., Solomon K.R., Freeman M.R. (2005). Cholesterol targeting alters lipid raft composition and cell survival in prostate cancer cells and xenografts. J. Clin. Investig..

[84] Solomon K.R., Pelton K., Boucher K., Joo J., Tully C., Zurakowski D., Schaffner C.P., Kim J., Freeman M.R. (2009). Ezetimibe Is an Inhibitor of Tumor Angiogenesis. Am. J. Pathol..

[85] Mostaghel E.A., Solomon K.R., Pelton K., Freeman M.R., Montgomery R.B. (2012). Impact of Circulating Cholesterol Levels on Growth and Intratumoral Androgen Concentration of Prostate Tumors. PLoS ONE.

[86] Dłubek J., Rysz J., Jabłonowski Z., Gluba-Brzózka A., Franczyk B. (2021). The Correlation between Lipid Metabolism Disorders and Prostate Cancer. Curr. Med. Chem..

[87] Cai C., Balk S.P. (2011). Intratumoral androgen biosynthesis in prostate cancer pathogenesis and response to therapy. Endocr.-Relat. Cancer.

[88] Deb S., Pham S., Ming D.-S., Chin M.Y., Adomat H., Hurtado-Coll A., Gleave M.E., Guns E.S.T. (2018). Characterization of Precursor-Dependent Steroidogenesis in Human Prostate Cancer Models. Cancers.

[89] Chen Y., Hughes-Fulford M. (2001). Human prostate cancer cells lack feedback regulation of low-density lipoprotein receptor and its regulator, SREBP2. Int. J. Cancer.

[90] Schörghofer D., Kinslechner K., Preitschopf A., Schütz B., Röhrl C., Hengstschläger M., Stangl H., Mikula M. (2015). The HDL receptor SR-BI is associated with human prostate cancer progression and plays a possible role in establishing androgen independence. Reprod. Biol. Endocrinol..

[91] Li-Beisson Y., Carrière F. (2020). Biogenesis and fate of lipid droplets. Biochimie.

[92] Lee B., Taylor M., Robinet P., Smith J.D., Schweitzer J., Sehayek E., Falzarano S.M., Magi-Galluzzi C., Klein E.A., Ting A.H. (2013). Dysregulation of Cholesterol Homeostasis in Human Prostate Cancer through Loss of ABCA1. Cancer Res..

[93] Murtola T.J., Syvälä H., Pennanen P., Bläuer M., Solakivi T., Ylikomi T., Tammela T.L.J. (2012). The Importance of LDL and Cholesterol Metabolism for Prostate Epithelial Cell Growth. PLoS ONE.

[94] Sekine Y., Demosky S.J., Stonik J.A., Furuya Y., Koike H., Suzuki K., Remaley A.T. (2010). High-Density Lipoprotein Induces Proliferation and Migration of Human Prostate Androgen–Independent Cancer Cells by an ABCA1-Dependent Mechanism. Mol. Cancer Res..

[95] Liu Y., Wang Y., Hao S., Qin Y., Wu Y. (2021). Knockdown of sterol O-acyltransferase 1 (SOAT1) suppresses SCD1-mediated lipogenesis and cancer procession in prostate cancer. Prostaglandins Other Lipid Mediat..

[96] Zhang D., Lagace T.A., Garuti R., Zhao Z., McDonald M., Horton J.D., Cohen J.C., Hobbs H.H. (2007). Binding of Proprotein Convertase Subtilisin/Kexin Type 9 to Epidermal Growth Factor-like Repeat A of Low Density Lipoprotein Receptor Decreases Receptor Recycling and Increases Degradation. J. Biol. Chem..

[97] Mahboobnia K., Pirro M., Marini E., Grignani F., Bezsonov E.E., Jamialahmadi T., Sahebkar A. (2021). PCSK9 and cancer: Rethinking the link. Biomed. Pharmacother..

[98] Klein-Szanto A.J., Bassi D.E. (2017). Proprotein convertase inhibition: Paralyzing the cell’s master switches. Biochem. Pharmacol..

[99] Jernberg E., Bergh A., Wikström P. (2017). Clinical relevance of androgen receptor alterations in prostate cancer. Endocr. Connect..

[100] Alaupovic P., Heinonen T., Shurzinske L., Black D.M. (1997). Effect of a new HMG-CoA reductase inhibitor, atorvastatin, on lipids, apolipoproteins and lipoprotein particles in patients with elevated serum cholesterol and triglyceride levels. Atherosclerosis.

[101] Olsson A.G., Pears J., McKellar J., Mizan J., Raza A. (2001). Effect of rosuvastatin on low-density lipoprotein cholesterol in patients with hypercholesterolemia. Am. J. Cardiol..

[102] Giugliano R.P., Desai N.R., Kohli P., Rogers W.J., Somaratne R., Huang F., Liu T., Mohanavelu S., Hoffman E.B., McDonald S.T. (2012). Efficacy, safety, and tolerability of a monoclonal antibody to proprotein convertase subtilisin/kexin type 9 in combination with a statin in patients with hypercholesterolaemia (LAPLACE-TIMI 57): A randomised, placebo-controlled, dose-ranging, phase 2 study. Lancet.

[103] Chan J.C.Y., Piper D.E., Cao Q., Liu D., King C., Wang W., Tang J., Liu Q., Higbee J., Xia Z. (2009). A proprotein convertase subtilisin/kexin type 9 neutralizing antibody reduces serum cholesterol in mice and nonhuman primates. Proc. Natl. Acad. Sci. USA.

[104] McCague R., Rowlands M.G., Barrie S.E., Houghton J. (1990). Inhibition of enzymes of estrogen and androgen biosynthesis by esters of 4-pyridylacetic acid. J. Med. Chem..

[105] Baigent C., Keech A.C., Kearney P., Blackwell L., Buck G., Pollicino C., Kirby A., Sourjina T., Peto R., Collins R. (2005). Efficacy and safety of cholesterol-lowering treatment: Prospective meta-analysis of data from 90,056 participants in 14 randomised trials of statins. Lancet.

[106] Baigent C., Blackwell L., Emberson J., Holland L.E., Reith C., Bhala N., Peto R., Barnes E.H., Keech A., Cholesterol Treatment Trialists’ (CTT) Collaboration (2010). Efficacy and safety of more intensive lowering of LDL cholesterol: A meta-analysis of data from 170,000 participants in 26 randomised trials. Lancet.

[107] Fulcher J., O’Connell R., Voysey M., Emberson J., Blackwell L., Mihaylova B., Simes J., Collins R., Kirby A., Cholesterol Treatment Trialists’ (CTT) Collaboration (2015). Efficacy and safety of LDL-lowering therapy among men and women: Meta-analysis of individual data from 174,000 participants in 27 randomised trials. Lancet.

[108] Barbalata C.I., Tefas L.R., Achim M., Tomuta I., Porfire A.S. (2020). Statins in risk-reduction and treatment of cancer. World J. Clin. Oncol..

[109] Stancu C.S., Sima A. (2001). Statins: Mechanism of action and effects. J. Cell. Mol. Med..

[110] Feingold K.R. (2000). Cholesterol Lowering Drugs.

[111] Ferri N., Corsini A. (2020). Clinical Pharmacology of Statins: An Update. Curr. Atheroscler. Rep..

[112] Ikonen E. (2008). Cellular cholesterol trafficking and compartmentalization. Nat. Rev. Mol. Cell Biol..

[113] Rached F., Santos R.D. (2020). The Role of Statins in Current Guidelines. Curr. Atheroscler. Rep..

[114] Demierre M.-F., Higgins P.D.R., Gruber S.B., Hawk E.T., Lippman S.M. (2005). Statins and cancer prevention. Nat. Rev. Cancer.

[115] Papadopoulos G., Delakas D., Nakopoulou L., Kasimatis T. (2011). Statins and prostate cancer: Molecular and clinical aspects. Eur. J. Cancer.

[116] Alfaqih M.A., Allott E.H., Hamilton R.J., Freeman M.R., Freedland S.J. (2017). The current evidence on statin use and prostate cancer prevention: Are we there yet?. Nat. Rev. Urol..

[117] Mucci L.A., Stampfer M.J. (2014). Mounting Evidence for Prediagnostic Use of Statins in Reducing Risk of Lethal Prostate Cancer. J. Clin. Oncol..

[118] Hatano K., Fujita K., Nonomura N. (2020). Application of Anti-Inflammatory Agents in Prostate Cancer. J. Clin. Med..

[119] Hoque A., Chen H., Xu X.-C. (2008). Statin Induces Apoptosis and Cell Growth Arrest in Prostate Cancer Cells. Cancer Epidemiol. Biomark. Prev..

[120] Brown M., Hart C.A., Tawadros T., Ramani V., Sangar V., Lau M., Clarke N. (2012). The differential effects of statins on the metastatic behaviour of prostate cancer. Br. J. Cancer.

[121] Ingersoll M.A., Miller D.R., Martinez O., Wakefield C.B., Hsieh K.-C., Simha M.V., Kao C.-L., Chen H.-T., Batra S.K., Lin M.-F. (2016). Statin derivatives as therapeutic agents for castration-resistant prostate cancer. Cancer Lett..

[122] Neuwirt H., Bouchal J., Kharaishvili G., Ploner C., Jöhrer K., Pitterl F., Weber A., Klocker H., Eder I.E. (2020). Cancer-associated fibroblasts promote prostate tumor growth and progression through upregulation of cholesterol and steroid biosynthesis. Cell Commun. Signal..

[123] Syvälä H., Pennanen P., Bläuer M., Tammela T.L., Murtola T.J. (2016). Additive inhibitory effects of simvastatin and enzalutamide on androgen-sensitive LNCaP and VCaP prostate cancer cells. Biochem. Biophys. Res. Commun..

[124] Wang H., Cui X.-X., Goodin S., Ding N., Van Doren J., Du Z., Huang M.-T., Liu Y., Cheng X., Dipaola R.S. (2013). Inhibition of IL-6 expression in LNCaP prostate cancer cells by a combination of atorvastatin and celecoxib. Oncol. Rep..

[125] Parikh A., Childress C., Deitrick K., Lin Q., Rukstalis D., Yang W. (2010). Statin-induced autophagy by inhibition of geranylgeranyl biosynthesis in prostate cancer PC3 cells. Prostate.

[126] Zheng X., Cui X.-X., Gao Z., Zhao Y., Lin Y., Shih W.J., Huang M.-T., Liu Y., Rabson A., Reddy B. (2010). Atorvastatin and Celecoxib in Combination Inhibits the Progression of Androgen-Dependent LNCaP Xenograft Prostate Tumors to Androgen Independence. Cancer Prev. Res..

[127] Krycer J.R., Kristiana I., Brown A.J. (2009). Cholesterol Homeostasis in Two Commonly Used Human Prostate Cancer Cell-Lines, LNCaP and PC-3. PLoS ONE.

[128] Yang L., Egger M., Plattner R., Klocker H., Eder I.E. (2011). Lovastatin Causes Diminished PSA Secretion by Inhibiting AR Expression and Function in LNCaP Prostate Cancer Cells. Urology.

[129] Graaf M.R., Beiderbeck A.B., Egberts T., Richel D.J., Guchelaar H.-J. (2004). The Risk of Cancer in Users of Statins. J. Clin. Oncol..

[130] Friis S., Poulsen A.H., Johnsen S.P., McLaughlin J.K., Fryzek J.P., Dalton S.O., Sørensen H.T., Olsen J.H. (2005). Cancer risk among statin users: A population-based cohort study. Int. J. Cancer.

[131] Shannon J., Tewoderos S., Garzotto M., Beer T.M., Derenick R., Palma A., Farris P.E. (2005). Statins and Prostate Cancer Risk: A Case-Control Study. Am. J. Epidemiol..

[132] Singal R., Khurana V., Caldito G., Fort C. (2005). Statins and prostate cancer risk: A large case control study in veterans. J. Clin. Oncol..

[133] Platz E.A., Leitzmann M.F., Visvanathan K., Rimm E.B., Stampfer M.J., Willett W.C., Giovannucci E. (2006). Statin Drugs and Risk of Advanced Prostate Cancer. J. Natl. Cancer Inst..

[134] Murtola T.J., Tammela T.L., Lahtela J., Auvinen A. (2007). Cholesterol-Lowering Drugs and Prostate Cancer Risk: A Population-based Case-Control Study. Cancer Epidemiol. Biomark. Prev..

[135] Jacobs E.J., Rodriguez C., Bain E.B., Wang Y., Thun M.J., Calle E.E. (2007). Cholesterol-Lowering Drugs and Advanced Prostate Cancer Incidence in a Large U.S. Cohort. Cancer Epidemiol. Biomark. Prev..

[136] Bonovas S., Filioussi K., Sitaras N.M. (2008). Statin use and the risk of prostate cancer: A metaanalysis of 6 randomized clinical trials and 13 observational studies. Int. J. Cancer.

[137] Breau R.H., Karnes R.J., Jacobson D.J., McGree M.E., Jacobsen S.J., Nehra A., Lieber M.M., Sauver J.L.S. (2010). The Association Between Statin Use and the Diagnosis of Prostate Cancer in a Population Based Cohort. J. Urol..

[138] Murtola T.J., Tammela T.L., Määttänen L., Huhtala H., Platz E.A., Ala-Opas M., Stenman U.-H., Auvinen A. (2010). Prostate cancer and PSA among statin users in the Finnish prostate cancer screening trial. Int. J. Cancer.

[139] Farwell W.R., D’Avolio L.W., Scranton R.E., Lawler E.V., Gaziano J.M. (2011). Statins and Prostate Cancer Diagnosis and Grade in a Veterans Population. J. Natl. Cancer Inst..

[140] Tan N., Klein E.A., Li J., Moussa A.S., Jones J.S. (2011). Statin Use and Risk of Prostate Cancer in a Population of Men Who Underwent Biopsy. J. Urol..

[141] Bansal D., Undela K., D’Cruz S., Schifano F. (2012). Statin Use and Risk of Prostate Cancer: A Meta-Analysis of Observational Studies. PLoS ONE.

[142] Jespersen C.G., Nørgaard M., Friis S., Skriver C., Borre M. (2014). Statin use and risk of prostate cancer: A Danish population-based case-control study, 1997–2010. Cancer Epidemiol..

[143] Lustman A., Nakar S., Cohen A.D., Vinker S. (2014). Statin use and incident prostate cancer risk: Does the statin brand matter? A population-based cohort study. Prostate Cancer Prostatic Dis..

[144] Van Rompay M.I., Solomon K.R., Nickel J.C., Ranganathan G., Kantoff P.W., McKinlay J.B. (2019). Prostate cancer incidence and mortality among men using statins and non-statin lipid-lowering medications. Eur. J. Cancer.

[145] Allott E.H., Ebot E.M., Stopsack K.H., Gonzalez-Feliciano A.G., Markt S.C., Wilson K.M., Ahearn T.U., Gerke T., Downer M.K., Rider J.R. (2020). Statin Use Is Associated with Lower Risk of PTEN-Null and Lethal Prostate Cancer. Clin. Cancer Res..

[146] Kantor E.D., Lipworth L., Fowke J.H., Giovannucci E.L., Mucci L.A., Signorello L.B. (2015). Statin use and risk of prostate cancer: Results from the Southern Community Cohort Study. Prostate.

[147] Park H.S., Schoenfeld J.D., Mailhot R.B., Shive M., Hartman R.I., Ogembo R., Mucci L.A. (2013). Statins and prostate cancer recurrence following radical prostatectomy or radiotherapy: A systematic review and meta-analysis. Ann. Oncol..

[148] Geybels M.S., Wright J.L., Holt S.K., Kolb S., Feng Z., Stanford J.L. (2013). Statin Use in Relation to Prostate Cancer Outcomes in a Population-based Patient Cohort Study. Prostate.

[149] Harshman L.C., Wang X., Nakabayashi M., Xie W., Valenca L.B., Werner L., Yu Y., Kantoff A.M., Sweeney C.J., Mucci L.A. (2015). Statin Use at the Time of Initiation of Androgen Deprivation Therapy and Time to Progression in Patients with Hormone-Sensitive Prostate Cancer. JAMA Oncol..

[150] Marcella S.W., David A., Ohman-Strickland P.A., Carson J., Rhoads G.G. (2012). Statin use and fatal prostate cancer: A matched case-control study. Cancer.

[151] Nielsen S.F., Nordestgaard B.G., Bojesen S.E. (2012). Statin Use and Reduced Cancer-Related Mortality. N. Engl. J. Med..

[152] Grytli H.H., Fagerland M.W., Fosså S.D., Taskén K.A. (2014). Association between use of beta-blockers and prostate cancer-specific survival: A cohort study of 3561 prostate cancer patients with high-risk or metastatic disease. Eur. Urol..

[153] Yu O., Eberg M., Benayoun S., Aprikian A., Batist G., Suissa S., Azoulay L. (2014). Use of Statins and the Risk of Death in Patients With Prostate Cancer. J. Clin. Oncol..

[154] Larsen S.B., Dehlendorff C., Skriver C., Dalton S.O., Jespersen C.G., Borre M., Brasso K., Nørgaard M., Johansen C., Sørensen H.T. (2017). Postdiagnosis Statin Use and Mortality in Danish Patients with Prostate Cancer. J. Clin. Oncol..

[155] Friedman G.D., Flick E.D., Udaltsova N., Chan Pharm D.J., Quesenberry C.P., Habel L. (2008). Screening statins for possible carcinogenic risk: Up to 9 years of follow-up of 361 859 recipients. Pharmacoepidemiol. Drug Saf..

[156] Kuoppala J., Lamminpää A., Pukkala E. (2008). Statins and cancer: A systematic review and meta-analysis. Eur. J. Cancer.

[157] Smeeth L., Douglas I., Hall A.J., Hubbard R., Evans S. (2009). Effect of statins on a wide range of health outcomes: A cohort study validated by comparison with randomized trials. Br. J. Clin. Pharmacol..

[158] Haukka J., Sankila R., Klaukka T., Lonnqvist J., Niskanen L., Tanskanen A., Wahlbeck K., Tiihonen J. (2010). Incidence of cancer and statin usage-Record linkage study. Int. J. Cancer.

[159] Hippisley-Cox J., Coupland C. (2010). Unintended effects of statins in men and women in England and Wales: Population based cohort study using the QResearch database. BMJ.

[160] Jacobs E.J., Newton C.C., Thun M.J., Gapstur S.M. (2011). Long-term Use of Cholesterol-Lowering Drugs and Cancer Incidence in a Large United States Cohort. Cancer Res..

[161] Chan J.M., Litwack-Harrison S., Bauer S., Daniels N.A., Wilt T.J., Shannon J., Bauer D.C. (2012). Statin Use and Risk of Prostate Cancer in the Prospective Osteoporotic Fractures in Men (MrOS) Study. Cancer Epidemiol. Biomark. Prev..

[162] Freedland S.J., Hamilton R., Gerber L., Banez L.L., Moreira D., Andriole G.L., Rittmaster R.S. (2013). Statin use and risk of prostate cancer and high-grade prostate cancer: Results from the REDUCE study. Prostate Cancer Prostatic Dis..

[163] Platz E.A., Tangen C.M., Goodman P.J., Till C., Parnes H.L., Figg W.D., Albanes D., Neuhouser M.L., Klein E.A., Lucia M.S. (2014). Statin Drug Use is Not Associated with Prostate Cancer Risk in Men Who are Regularly Screened. J. Urol..

[164] Fowke J.H., Motley S.S., Barocas D.A., Cookson M.S., Concepcion R., Byerly S., Smith J.A. (2011). The associations between statin use and prostate cancer screening, prostate size, high-grade prostatic intraepithelial neoplasia (PIN), and prostate cancer. Cancer Causes Control..

[165] Coogan P.F., Kelly J.P., Strom B.L., Rosenberg L. (2010). Statin and NSAID use and prostate cancer risk. Pharmacoepidemiol. Drug Saf..

[166] Boudreau D.M., Yu O., Buist D.S.M., Miglioretti D.L. (2008). Statin use and prostate cancer risk in a large population-based setting. Cancer Causes Control..

[167] Chang C.-C., Ho S.-C., Chiu H.-F., Yang C.-Y. (2011). Statins increase the risk of prostate cancer: A population-based case-control study. Prostate.

[168] Flick E.D., Habel L.A., Chan K.A., Van Den Eeden S.K., Quinn V.P., Haque R., Orav E.J., Seeger J.D., Sadler M.C., Quesenberry C.P. (2007). Statin use and risk of prostate cancer in the California Men’s Health Study cohort. Cancer Epidemiol. Biomark. Prev..

[169] Yanai Y., Kosaka T., Hongo H., Oya M. (2019). Clinically complete response to abiraterone acetate in a patient with metastatic castration-resistant prostate cancer. IJU Case Rep..

[170] Di Lorenzo G., Sonpavde G., Pond G., Lucarelli G., Rossetti S., Facchini G., Scagliarini S., Cartenì G., Federico P., Daniele B. (2018). Statin Use and Survival in Patients with Metastatic Castration-resistant Prostate Cancer Treated with Abiraterone Acetate. Eur. Urol. Focus.

[171] Yang H., Pang L., Hu X., Wang W., Xu B., Zhang X., Liu L. (2020). The effect of statins on advanced prostate cancer patients with androgen deprivation therapy or abiraterone/enzalutamide: A systematic review and meta-analysis. J. Clin. Pharm. Ther..

[172] Gordon J.A., Buonerba C., Pond G., Crona D., Gillessen S., Lucarelli G., Rossetti S., Dorff T., Artale S., Locke J.A. (2018). Statin use and survival in patients with metastatic castration-resistant prostate cancer treated with abiraterone or enzalutamide after docetaxel failure: The international retrospective observational STABEN study. Oncotarget.

[173] Gordon J.A., Midha A., Szeitz A., Ghaffari M., Adomat H.H., Guo Y., Klassen T.L., Guns E.S., Wasan K.M., Cox M.E. (2016). Oral simvastatin administration delays castration-resistant progression and reduces intratumoral steroidogenesis of LNCaP prostate cancer xenografts. Prostate Cancer Prostatic Dis..

[174] Twiddy A.L., Leon C.G., Wasan K.M. (2011). Cholesterol as a Potential Target for Castration-Resistant Prostate Cancer. Pharm. Res..

[175] Kim J.H., Cox M.E., Wasan K.M. (2014). Effect of simvastatin on castration-resistant prostate cancer cells. Lipids Health Dis..

[176] Zani I.A., Stephen S.L., Mughal N.A., Russell D., Homer-Vanniasinkam S., Wheatcroft S.B., Ponnambalam S. (2015). Scavenger Receptor Structure and Function in Health and Disease. Cells.

[177] Landschulz K.T., Pathak R.K., Rigotti A., Krieger M., Hobbs H.H. (1996). Regulation of scavenger receptor, class B, type I, a high density lipoprotein receptor, in liver and steroidogenic tissues of the rat. J. Clin. Investig..

[178] Rigotti A., Edelman E.R., Seifert P., Iqbal S.N., DeMattos R.B., Temel R.E., Krieger M., Williams D.L. (1996). Regulation by Adrenocorticotropic Hormone of the in Vivo Expression of Scavenger Receptor Class B Type I (SR-BI), a High Density Lipoprotein Receptor, in Steroidogenic Cells of the Murine Adrenal Gland. J. Biol. Chem..

[179] Canton J., Neculai D., Grinstein S. (2013). Scavenger receptors in homeostasis and immunity. Nat. Rev. Immunol..

[180] Shen W.-J., Azhar S., Kraemer F. (2018). SR-B1: A Unique Multifunctional Receptor for Cholesterol Influx and Efflux. Annu. Rev. Physiol..

[181] Krieger M. (1999). Charting the Fate of the “Good Cholesterol”: Identification and Characterization of the High-Density Lipoprotein Receptor SR-BI. Annu. Rev. Biochem..

[182] Rigotti A., Miettinen H.E., Krieger M. (2003). The Role of the High-Density Lipoprotein Receptor SR-BI in the Lipid Metabolism of Endocrine and Other Tissues. Endocr. Rev..

[183] Gwynne J.T., Mahaffee D.D. (1989). Rat adrenal uptake and metabolism of high density lipoprotein cholesteryl ester. J. Biol. Chem..

[184] Gwynne J.T., Mahaffee D., Brewer H.B., Ney R.L. (1976). Adrenal cholesterol uptake from plasma lipoproteins: Regulation by corticotropin. Proc. Natl. Acad. Sci. USA.

[185] Shen J.W., Azhar S., Kraemer F.B. (2016). ACTH Regulation of Adrenal SR-B1. Front. Endocrinol..

[186] Shen W.-J., Hu J., Hu Z., Kraemer F.B., Azhar S. (2014). Scavenger Receptor class B type I (SR-BI): A versatile receptor with multiple functions and actions. Metabolism.

[187] Gwynne J.T., Hess B., Hughes T., Rountree R., Mahaffee D. (1985). The Role of Serum High Density Lipoproteins in Adrenal Steroidogenesis. Endocr. Res..

[188] Gaälman C., Angelin B., Rudling M. (2002). Prolonged Stimulation of the Adrenals by Corticotropin Suppresses Hepatic Low-Density Lipoprotein and High-Density Lipoprotein Receptors and Increases Plasma Cholesterol. Endocrinology.

[189] Beaven S.W., Tontonoz P. (2006). Nuclear Receptors in Lipid Metabolism: Targeting the Heart of Dyslipidemia. Annu. Rev. Med..

[190] Cao G.P., Garcia C.K., Wyne K.L., Schultz R.A., Parker K.L., Hobbs H.H. (1997). Structure and localization of the human gene encoding SR-BI/CLA-1—Evidence for transcriptional control by steroidogenic factor 1. J. Biol. Chem..

[191] Parker K.L. (1998). The roles of steroidogenic factor 1 in endocrine development and function. Mol. Cell. Endocrinol..

[192] Lekstrom-Himes J., Xanthopoulos K.G. (1998). Biological Role of the CCAAT/Enhancer-binding Protein Family of Transcription Factors. J. Biol. Chem..

[193] Vickers K.C., Rodriguez A. (2014). Human scavenger receptor class B type I variants, lipid traits, and cardiovascular disease. Circ. Cardiovasc. Genet..

[194] Christianson M., Yates M. (2012). Scavenger receptor class B type 1 gene polymorphisms and female fertility. Curr. Opin. Endocrinol. Diabetes Obes..

[195] Valacchi G., Sticozzi C., Lim Y., Pecorelli A. (2011). Scavenger receptor class B type I: A multifunctional receptor. Ann. N. Y. Acad. Sci..

[196] Ma B., Jia J., Wang X., Zhang R., Niu S., Ni L., Di X., Liu C. (2020). Differential roles of Scavenger receptor class B type I: A protective molecule and a facilitator of atherosclerosis (Review). Mol. Med. Rep..

[197] Zheng Z., Ai J., Li X.-A. (2014). Scavenger receptor class B type I and immune dysfunctions. Curr. Opin. Endocrinol. Diabetes Obes..

[198] Shen W.-J., Asthana S., Kraemer F.B., Azhar S. (2018). Thematic Review Series: Lipid Transfer Proteins Scavenger receptor B type 1: Expression, molecular regulation, and cholesterol transport function. J. Lipid Res..

[199] Thi V.L.D., Dreux M., Cosset F.-L. (2011). Scavenger receptor class B type I and the hypervariable region-1 of hepatitis C virus in cell entry and neutralisation. Expert Rev. Mol. Med..

[200] Westhaus S., Deest M., Nguyen A.T., Stanke F., Heckl D., Costa R., Schambach A., Manns M.P., Berg T., Vondran F.W. (2017). Scavenger receptor class B member 1 (SCARB1) variants modulate hepatitis C virus replication cycle and viral load. J. Hepatol..

[201] Wei W.C., Wan L., Yan Q., Wang X., Zhang J., Yang X., Zhang Y., Fan C., Li D., Deng Y. (2020). HDL-scavenger receptor B type 1 facilitates SARS-CoV-2 entry. Nat. Metab..

[202] Thuahnai S.T., Lund-Katz S., Williams D.L., Phillips M.C. (2001). Scavenger receptor class B, type I-mediated uptake of various lipids into cells. Influence of the nature of the donor particle interaction with the receptor. J. Biol. Chem..

[203] Glass C., Pittman R.C., Weinstein D.B., Steinberg D. (1983). Dissociation of tissue uptake of cholesterol ester from that of apoprotein A-I of rat plasma high density lipoprotein: Selective delivery of cholesterol ester to liver, adrenal, and gonad. Proc. Natl. Acad. Sci. USA.

[204] Silver D.L., Wang N., Xiao X., Tall A.R. (2001). High Density Lipoprotein (HDL) Particle Uptake Mediated by Scavenger Receptor Class B Type 1 Results in Selective Sorting of HDL Cholesterol from Protein and Polarized Cholesterol Secretion. J. Biol. Chem..

[205] Gillard B.K., Bassett G.R., Gotto A.M., Rosales C., Pownall H. (2017). Scavenger receptor B1 (SR-B1) profoundly excludes high density lipoprotein (HDL) apolipoprotein AII as it nibbles HDL-cholesteryl ester. J. Biol. Chem..

[206] Irene G.-R., César M., Fernando C., Ana C. (2021). SR-B1, a Key Receptor Involved in the Progression of Cardiovascular Disease: A Perspective from Mice and Human Genetic Studies. Biomedicines.

[207] Neculai D., Schwake M., Ravichandran M., Zunke F., Collins R.F., Peters J., Neculai M., Plumb J., Loppnau P., Pizarro J.C. (2013). Structure of LIMP-2 provides functional insights with implications for SR-BI and CD36. Nature.

[208] Yancey P.G., de la Llera-Moya M., Swarnakar S., Monzo P., Klein S.M., Connelly M.A., Johnson W.J., Williams D.L., Rothblat G.H. (2000). High Density Lipoprotein Phospholipid Composition Is a Major Determinant of the Bi-directional Flux and Net Movement of Cellular Free Cholesterol Mediated by Scavenger Receptor BI. J. Biol. Chem..

[209] Reaven E., Chen Y.D., Spicher M., Azhar S. (1984). Morphological evidence that high density lipoproteins are not internalized by steroid-producing cells during in situ organ perfusion. J. Clin. Investig..

[210] Azhar S., Reaven E. (2002). Scavenger receptor class BI and selective cholesteryl ester uptake: Partners in the regulation of steroidogenesis. Mol. Cell. Endocrinol..

[211] Mineo C., Yuhanna I.S., Quon M., Shaul P.W. (2003). High Density Lipoprotein-induced Endothelial Nitric-oxide Synthase Activation Is Mediated by Akt and MAP Kinases. J. Biol. Chem..

[212] Wood P., Mulay V., Darabi M., Chan K.C., Heeren J., Pol A., Lambert G., Rye K.-A., Enrich C., Grewal T. (2011). Ras/Mitogen-activated Protein Kinase (MAPK) Signaling Modulates Protein Stability and Cell Surface Expression of Scavenger Receptor SR-BI. J. Biol. Chem..

[213] Grewal T., de Diego I., Kirchhoff M.F., Tebar F., Heeren J., Rinninger F., Enrich C. (2003). High Density Lipoprotein-induced Signaling of the MAPK Pathway Involves Scavenger Receptor Type BI-mediated Activation of Ras. J. Biol. Chem..

[214] Al-Jarallah A., Trigatti B.L. (2010). A role for the scavenger receptor, class B type I in high density lipoprotein dependent activation of cellular signaling pathways. Biochim. Biophys. Acta.

[215] Li X.-A., Titlow W.B., Jackson B.A., Giltiay N., Nikolova-Karakashian M., Uittenbogaard A., Smart E.J. (2002). High Density Lipoprotein Binding to Scavenger Receptor, Class B, Type I Activates Endothelial Nitric-oxide Synthase in a Ceramide-dependent Manner. J. Biol. Chem..

[216] Mineo C., Shaul P.W. (2003). HDL stimulation of endothelial nitric oxide synthase—A novel mechanism of HDL action. Trends Cardiovasc. Med..

[217] Yuhanna I.S., Zhu Y., Cox B.E., Hahner L.D., Osborne-Lawrence S., Lu P., Marcel Y.L., Anderson R.G., Mendelsohn M.E., Hobbs H.H. (2001). High-density lipoprotein binding to scavenger receptor-BI activates endothelial nitric oxide synthase. Nat. Med..

[218] Förstermann U., Xia N., Li H. (2017). Roles of Vascular Oxidative Stress and Nitric Oxide in the Pathogenesis of Atherosclerosis. Circ. Res..

[219] Tousoulis D., Kampoli A.-M., Papageorgiou N., Stefanadis C. (2012). The Role of Nitric Oxide on Endothelial Function. Curr. Vasc. Pharmacol..

[220] Li X.-A., Guo L., Dressman J.L., Asmis R., Smart E.J. (2005). A Novel Ligand-independent Apoptotic Pathway Induced by Scavenger Receptor Class B, Type I and Suppressed by Endothelial Nitric-oxide Synthase and High Density Lipoprotein. J. Biol. Chem..

[221] Nofer J.R., van der Giet M., Tölle M., Wolinska I., von Wnuck Lipinski K., Baba H.A., Tietge U.J., Gödecke A., Ishii I., Kleuser B. (2004). HDL induces NO-dependent vasorelaxation via the lysophospholipid receptor S1P3. J. Clin. Investig..

[222] Murata N., Sato K., Kon J., Tomura H., Yanagita M., Kuwabara A., Ui M., Okajima F. (2000). Interaction of sphingosine 1-phosphate with plasma components, including lipoproteins, regulates the lipid receptor-mediated actions. Biochem. J..

[223] Igarashi J., Michel T. (2001). Sphingosine 1-phosphate and isoform-specific activation of phosphoinositide 3-kinase beta. Evidence for divergence and convergence of receptor-regulated endothelial nitric-oxide synthase signaling pathways. J. Biol. Chem..

[224] Lorbek G., Lewinska M., Rozman D. (2012). Cytochrome P450s in the synthesis of cholesterol and bile acids—From mouse models to human diseases. FEBS J..

[225] Zhang W., Yancey P.G., Su Y.R., Babaev V.R., Zhang Y., Fazio S., Linton M.F. (2003). Inactivation of Macrophage Scavenger Receptor Class B Type I Promotes Atherosclerotic Lesion Development in Apolipoprotein E–Deficient Mice. Circulation.

[226] Tao H., Yancey P.G., Babaev V.R., Blakemore J.L., Zhang Y., Ding L., Fazio S., Linton M.F. (2015). Macrophage SR-BI mediates efferocytosis via Src/PI3K/Rac1 signaling and reduces atherosclerotic lesion necrosis. J. Lipid Res..

[227] Kruth H.S. (2001). Macrophage foam cells and atherosclerosis. Front. Biosci..

[228] Tall R.A., Costet P., Wang N. (2002). Regulation and mechanisms of macrophage cholesterol efflux. J. Clin. Investig..

[229] Yancey P.G., Bortnick A.E., Kellner-Weibel G., De La Llera-Moya M., Phillips M.C., Rothblat G.H. (2003). Importance of Different Pathways of Cellular Cholesterol Efflux. Arter. Thromb. Vasc. Biol..

[230] von Eckardstein A. (1996). Cholesterol efflux from macrophages and other cells. Curr. Opin. Lipidol..

[231] Ross R. (1999). Atherosclerosis is an inflammatory disease. Am. Heart J..

[232] Nieland T.J.F., Shaw J., Jaipuri F.A., Duffner J.L., Koehler A.N., Banakos S., Zannis V.I., Kirchhausen T., Krieger M. (2008). Identification of the Molecular Target of Small Molecule Inhibitors of HDL Receptor SR-BI Activity. Biochemistry.

[233] Nieland T.J.F., Penman M., Dori L., Krieger M., Kirchhausen T. (2002). Nonlinear partial differential equations and applications: Discovery of chemical inhibitors of the selective transfer of lipids mediated by the HDL receptor SR-BI. Proc. Natl. Acad. Sci. USA.

[234] Yu M., Romer K.A., Nieland T.J.F., Xu S., Saenz-Vash V., Penman M., Yesilaltay A., Carr S.A., Krieger M. (2011). Exoplasmic cysteine Cys384 of the HDL receptor SR-BI is critical for its sensitivity to a small-molecule inhibitor and normal lipid transport activity. Proc. Natl. Acad. Sci. USA.

[235] Nieland T.J.F., Chroni A., Fitzgerald M.L., Maliga Z., Zannis V., Kirchhausen T., Krieger M. (2004). Cross-inhibition of SR-BI- and ABCA1-mediated cholesterol transport by the small molecules BLT-4 and glyburide. J. Lipid Res..

[236] Masson D., Koseki M., Ishibashi M., Larson C.J., Miller S.G., King B.D., Tall A.R. (2009). Increased HDL cholesterol and apoA-I in humans and mice treated with a novel SR-BI inhibitor. Arterioscler. Thromb. Vasc. Biol..

[237] Rowe I.A., Tully D.C.D.C., Armstrong M.J., Parker R., Guo K., Barton D., Morse G.D.G.D., Venuto C.S.C.S., Ogilvie C.B., Hedegaard D.L. (2016). Effect of scavenger receptor class B type I antagonist ITX5061 in patients with hepatitis C virus infection undergoing liver transplantation. Liver Transplant..

[238] Sulkowski M.S., Kang M., Matining R., Wyles D., Johnson V.A., Morse G.D., Amorosa V., Bhattacharya D., Coughlin K., Wong-Staal F. (2014). Safety and Antiviral Activity of the HCV Entry Inhibitor ITX5061 in Treatment-Naive HCV-Infected Adults: A Randomized, Double-Blind, Phase 1b Study. J. Infect. Dis..

[239] Nishizawa T., Kitayama K., Wakabayashi K., Yamada M., Uchiyama M., Abe K., Ubukata N., Inaba T., Oda T., Amemiya Y. (2007). A novel compound, R-138329, increases plasma HDL cholesterol via inhibition of scavenger receptor BI-mediated selective lipid uptake. Atherosclerosis.

[240] Dockendorff C., Faloon P.W., Yu M., Youngsaye W., Penman M., Nieland T.J.F., Nag P.P., Lewis T., Pu J., Bennion M. (2015). Indolinyl-Thiazole Based Inhibitors of Scavenger Receptor-BI (SR-BI)-Mediated Lipid Transport. ACS Med. Chem. Lett..

[241] Faloon P.W., Dockendorff C., Youngsaye W., Yu M., Nag P.P., Lewis T.A., Bennion M., Paterson C., Lam G., Dandapani S. (2010). A Small Molecule Inhibitor of Scavenger Receptor BI-mediated Lipid Uptake—Probe 1. Probe Reports from the NIH Molecular Libraries Program.

[242] Dockendorff C., Faloon P.W., Germain A., Yu M., Youngsaye W., Nag P.P., Bennion M., Penman M., Nieland T.J.F., Dandapani S. (2015). Discovery of bisamide-heterocycles as inhibitors of scavenger receptor BI (SR-BI)-mediated lipid uptake. Bioorganic Med. Chem. Lett..

[243] Dockendorff C., Faloon P.W., Pu J., Yu M., Johnston S., Bennion M., Penman M., Nieland T.J., Dandapani S., Perez J.R. (2015). Benzo-fused lactams from a diversity-oriented synthesis (DOS) library as inhibitors of scavenger receptor BI (SR-BI)-mediated lipid uptake. Bioorgan. Med. Chem. Lett..

[244] Zheng Y., Liu Y., Jin H., Pan S., Qian Y., Huang C., Zeng Y., Luo Q., Zeng M., Zhang Z. (2013). Scavenger Receptor B1 is a Potential Biomarker of Human Nasopharyngeal Carcinoma and Its Growth is Inhibited by HDL-mimetic Nanoparticles. Theranostics.

[245] Julovi S.M., Xue A., Le T.N.T., Gill A.J., Bulanadi J.C., Patel M., Waddington L.J., Rye K.-A., Moghaddam M.J., Smith R.C. (2016). Apolipoprotein A-II Plus Lipid Emulsion Enhance Cell Growth via SR-B1 and Target Pancreatic Cancer In Vitro and In Vivo. PLoS ONE.

[246] McMahon K.M., Foit L., Angeloni N.L., Giles F.J., Gordon L.I., Thaxton C.S. (2015). Synthetic High-Density Lipoprotein-Like Nanoparticles as Cancer Therapy. Cancer Treat. Res..

[247] Berney E., Sabnis N., Panchoo M., Raut S., Dickerman R., Lacko A.G. (2019). The SR-B1 Receptor as a Potential Target for Treating Glioblastoma. J. Oncol..

[248] Leon C.G., Locke J.A., Adomat H.H., Etinger S.L., Twiddy A.L., Neumann R.D., Nelson C.C., Guns E.S., Wasan K.M. (2010). Alterations in cholesterol regulation contribute to the production of intratumoral androgens during progression to castration-resistant prostate cancer in a mouse xenograft model. Prostate.

[249] Twiddy A.L., Cox M.E., Wasan K.M. (2012). Knockdown of scavenger receptor Class B Type I reduces prostate specific antigen secretion and viability of prostate cancer cells. Prostate.

[250] Gordon J.A., Noble J.W., Midha A., Derakhshan F., Wang G., Adomat H.H., Guns E.S.T., Lin Y.-Y., Ren S., Collins C.C. (2019). Upregulation of Scavenger Receptor B1 Is Required for Steroidogenic and Nonsteroidogenic Cholesterol Metabolism in Prostate Cancer. Cancer Res..

[251] Patel R., Mui E., Loveridge C., Repiscak P., Ahmad I., Hamdy F.C., Leung H.Y. (2018). Targeting cholesterol transport in castration-resistant prostate cancer. Clin. Cancer Res..

[252] Traughber C.A., Opoku E., Brubaker G., Major J., Lu H., Lorkowski S.W., Neumann C., Hardaway A., Chung Y.-M., Gulshan K. (2020). Uptake of high-density lipoprotein by scavenger receptor class B type 1 is associated with prostate cancer proliferation and tumor progression in mice. J. Biol. Chem..

[253] Massie C., Lynch A., Ramos-Montoya A., Boren J., Stark R., Fazli L., Warren A., Scott H., Madhu B., Sharma N. (2011). The androgen receptor fuels prostate cancer by regulating central metabolism and biosynthesis. EMBO J..

[254] Culig Z., Santer F.R. (2014). Androgen receptor signaling in prostate cancer. Cancer Metastasis Rev..

[255] Giunchi F., Fiorentino M., Loda M. (2019). The Metabolic Landscape of Prostate Cancer. Eur. Urol. Oncol..

[256] Rocchi P., So A., Kojima S., Signaevsky M., Beraldi E., Fazli L., Hurtado-Coll A., Yamanaka K., Gleave M. (2004). Heat shock protein 27 increases after androgen ablation and plays a cytoprotective role in hormone-refractory prostate cancer. Cancer Res..

[257] Rocchi P., Jugpal P., So A., Sinneman S., Ettinger S., Fazli L., Nelson C., Gleave M. (2006). Small interference RNA targeting heat-shock protein 27 inhibits the growth of prostatic cell lines and induces apoptosis via caspase-3 activation in vitro. BJU Int..

[258] Zoubeidi A., Zardan A., Beraldi E., Fazli L., Sowery R., Rennie P., Nelson C., Gleave M. (2007). Cooperative Interactions between Androgen Receptor (AR) and Heat-Shock Protein 27 Facilitate AR Transcriptional Activity. Cancer Res..

[259] Zoubeidi A., Zardan A., Wiedmann R.M., Locke J., Beraldi E., Fazli L., Gleave M.E. (2010). Hsp27 Promotes Insulin-Like Growth Factor-I Survival Signaling in Prostate Cancer via p90Rsk-Dependent Phosphorylation and Inactivation of BAD. Cancer Res..

[260] Shiota M., Bishop J.L., Nip K.M., Zardan A., Takeuchi A., Cordonnier T., Beraldi E., Bazov J., Fazli L., Chi K. (2013). Hsp27 Regulates Epithelial Mesenchymal Transition, Metastasis, and Circulating Tumor Cells in Prostate Cancer. Cancer Res..

[261] Al Nakouzi N., Wang C.K., Beraldi E., Jager W., Ettinger S., Fazli L., Nappi L., Bishop J., Zhang F., Chauchereau A. (2016). Clusterin knockdown sensitizes prostate cancer cells to taxane by modulating mitosis. EMBO Mol. Med..

[262] Zhang F., Kumano M., Beraldi E., Fazli L., Du C., Moore S., Sorensen P., Zoubeidi A., Gleave M.E. (2014). Clusterin facilitates stress-induced lipidation of LC3 and autophagosome biogenesis to enhance cancer cell survival. Nat. Commun..

[263] Altman B., Rathmell J.C. (2012). Metabolic Stress in Autophagy and Cell Death Pathways. Cold Spring Harb. Perspect. Biol..

[264] Farrow J.M., Yang J.C., Evans C.P. (2014). Autophagy as a modulator and target in prostate cancer. Nat. Rev. Urol..

[265] Bennett H.L., Stockley J., Fleming J.T., Mandal R., O’Prey J., Ryan K.M., Robson C.N., Leung H.Y. (2013). Does androgen-ablation therapy (AAT) associated autophagy have a pro-survival effect in LNCaP human prostate cancer cells?. BJU Int..

[266] Nguyen H.G., Yang J.C., Kung H.-J., Shi X.-B., Tilki D., Lara P.N., White R.W.D., Gao A.C., Evans C.P. (2014). Targeting autophagy overcomes Enzalutamide resistance in castration-resistant prostate cancer cells and improves therapeutic response in a xenograft model. Oncogene.

[267] Chu Y., Chang Y., Lu W., Sheng X., Wang S., Xu H., Ma J. (2020). Regulation of Autophagy by Glycolysis in Cancer. Cancer Manag. Res..

[268] Hsu P.P., Kang S.A., Rameseder J., Zhang Y., Ottina K.A., Lim D., Peterson T.R., Choi Y., Gray N.S., Yaffe M.B. (2011). The mTOR-Regulated Phosphoproteome Reveals a Mechanism of mTORC1-Mediated Inhibition of Growth Factor Signaling. Science.

[269] Mihaylova M.M., Shaw R.J. (2011). The AMPK signalling pathway coordinates cell growth, autophagy and metabolism. Nature.

[270] McInnes K.J., Brown K., Hunger N.I., Simpson E.R. (2012). Regulation of LKB1 expression by sex hormones in adipocytes. Int. J. Obes..

[271] Lemos C., Schulze V.K., Baumgart S.J., Nevedomskaya E., Heinrich T., Lefranc J., Bader B., Christ C.D., Briem H., Kuhnke L.P. (2021). The potent AMPK inhibitor BAY-3827 shows strong efficacy in androgen-dependent prostate cancer models. Cell. Oncol..

[272] Hoyerhansen M., Jaattela M. (2007). Connecting endoplasmic reticulum stress to autophagy by unfolded protein response and calcium. Cell Death Differ..

[273] Metur S.P., Klionsky D.J. (2021). Adaptive immunity at the crossroads of autophagy and metabolism. Cell. Mol. Immunol..

[274] Li W., He P., Huang Y., Li Y.-F., Lu J., Li M., Kurihara H., Luo Z., Meng T., Onishi M. (2021). Selective autophagy of intracellular organelles: Recent research advances. Theranostics.

[275] Rashid H.-O., Yadav R.K., Kim H.-R., Chae H.-J. (2015). ER stress: Autophagy induction, inhibition and selection. Autophagy.

[276] Röhrl C., Stangl H. (2018). Cholesterol metabolism—Physiological regulation and pathophysiological deregulation by the endoplasmic reticulum. Wien. Med. Wochenschr..

[277] Melamed J., Datta M.W., Becich M.J., Orenstein J.M., Dhir R., Silver S., Fidélia-Lambert M., Kadjacsy-Balla A., Macias V., Patel A. (2004). The cooperative prostate cancer tissue resource: A specimen and data resource for cancer researchers. Clin. Cancer Res..

[278] Berman J.J., Datta M., Kajdacsy-Balla A., Melamed J., Orenstein J., Dobbin K., Patel A., Dhir R., Becich M.J. (2004). The tissue microarray data exchange specification: Implementation by the Cooperative Prostate Cancer Tissue Resource. BMC Bioinform..

[279] Dash R.C., Robb J.A., Booker D.L., Foo W.-C., Witte D.L., Bry L. (2012). Biospecimens and Biorepositories for the Community Pathologist. Arch. Pathol. Lab. Med..

[280] Wei B.-R., Simpson R.M. (2014). Digital pathology and image analysis augment biospecimen annotation and biobank quality assurance harmonization. Clin. Biochem..

[281] Gordon J.A. (2018). Cholesterol metabolism as a target in castration-resistant prostate cancer (T). Ph.D. Thesis.

[282] Liu L.L., Xie N., Sun S., Plymate S.R., Mostaghel E.A., Dong X. (2014). Mechanisms of the androgen receptor splicing in prostate cancer cells. Oncogene.

[283] Lai S.L., Brauch H., Knutsen T., Johnson B.E., Nau M.M., Mitsudomi T., Tsai C.M., Whang-Peng J., Zbar B., Kaye F.J. (1995). Molecular genetic characterization of neuroendocrine lung cancer cell lines. Anticancer Res..

[284] Kuruma H., Matsumoto H., Shiota M., Bishop J., Lamoureux F., Thomas C., Briere D., Los G., Gleave M., Fanjul A. (2013). A Novel Antiandrogen, Compound 30, Suppresses Castration-Resistant and MDV3100-Resistant Prostate Cancer Growth In Vitro and In Vivo. Mol. Cancer Ther..

[285] Mostaghel E.A., Marck B.T., Plymate S.R., Vessella R.L., Balk S., Matsumoto A.M., Nelson P.S., Montgomery R.B. (2011). Resistance to CYP17A1 Inhibition with Abiraterone in Castration-Resistant Prostate Cancer: Induction of Steroidogenesis and Androgen Receptor Splice Variants. Clin. Cancer Res..

[286] Daniel V.C., Marchionni L., Hierman J.S., Rhodes J.T., Devereux W.L., Rudin C., Yung R., Parmigiani G., Dorsch M., Peacock C.D. (2009). A Primary Xenograft Model of Small-Cell Lung Cancer Reveals Irreversible Changes in Gene Expression Imposed by Culture In vitro. Cancer Res..

[287] Tentler J.J., Tan A.C., Weekes C.D., Jimeno A., Leong S., Pitts T.M., Arcaroli J.J., Messersmith W.A., Eckhardt S.G. (2012). Patient-derived tumour xenografts as models for oncology drug development. Nat. Rev. Clin. Oncol..

[288] Nguyen H.M., Vessella R.L., Morrissey C., Brown L.G., Coleman I.M., Higano C.S., Mostaghel E.A., Zhang X., True L.D., Lam H.-M. (2017). LuCaP Prostate Cancer Patient-Derived Xenografts Reflect the Molecular Heterogeneity of Advanced Disease and Serve as Models for Evaluating Cancer Therapeutics. Prostate.

[289] Davies A.H., Wang Y., Zoubeidi A. (2018). Patient-derived xenografts: A platform for accelerating translational research in prostate cancer. Mol. Cell. Endocrinol..

[290] Oppi S., Luscher T.F., Stein S. (2019). Mouse Models for Atherosclerosis Research-Which Is My Line?. Front. Cardiovasc. Med..

[291] Shao W., Espenshade P.J. (2012). Expanding Roles for SREBP in Metabolism. Cell Metab..

[292] Subczynski W.K., Pasenkiewicz-Gierula M., Widomska J., Mainali L., Raguz M. (2017). High Cholesterol/Low Cholesterol: Effects in Biological Membranes: A Review. Cell Biophys..

[293] Sheng R., Chen Y., Gee H.Y., Stec E., Melowic H.R., Blatner N.R., Tun M.P., Kim Y., Källberg M., Fujiwara T. (2012). Cholesterol modulates cell signaling and protein networking by specifically interacting with PDZ domain-containing scaffold proteins. Nat. Commun..

[294] Beese C.J., Brynjólfsdóttir S.H., Frankel L.B. (2019). Selective Autophagy of the Protein Homeostasis Machinery: Ribophagy, Proteaphagy and ER-Phagy. Front. Cell Dev. Biol..

[295] Velázquez A.P., Graef M. (2016). Autophagy regulation depends on ER homeostasis controlled by lipid droplets. Autophagy.

[296] Ribas V., García-Ruiz C., Fernández-Checa J.C. (2016). Mitochondria, cholesterol and cancer cell metabolism. Clin. Transl. Med..

[297] Robichon C., Dugail I. (2007). De novo cholesterol synthesis at the crossroads of adaptive response to extracellular stress through SREBP. Biochimie.

[298] DeBose-Boyd R.A. (2008). Feedback regulation of cholesterol synthesis: Sterol-accelerated ubiquitination and degradation of HMG CoA reductase. Cell Res..

[299] de Laurentiis A., Donovan L., Arcaro A. (2007). Lipid rafts and caveolae in signaling by growth factor receptors. Open Biochem. J..

[300] Day K.C., Hiles G.L., Kozminsky M., Dawsey S.J., Paul A., Broses L., Shah R., Kunja L.P., Hall C., Palanisamy N. (2017). HER2 and EGFR Overexpression Support Metastatic Progression of Prostate Cancer to Bone. Cancer Res..

[301] Wu J., Yu E. (2014). Insulin-like growth factor receptor-1 (IGF-IR) as a target for prostate cancer therapy. Cancer Metastasis Rev..

[302] Mukherjee R., McGuinness D.H., McCall P., Underwood M.A., Seywright M., Orange C., Edwards J. (2011). Upregulation of MAPK pathway is associated with survival in castrate-resistant prostate cancer. Br. J. Cancer.

[303] Siddle K. (2012). Molecular Basis of Signaling Specificity of Insulin and IGF Receptors: Neglected Corners and Recent Advances. Front. Endocrinol..

[304] Shanware N.P., Bray K., Abraham R.T. (2013). The PI3K, Metabolic, and Autophagy Networks: Interactive Partners in Cellular Health and Disease. Annu. Rev. Pharmacol. Toxicol..

[305] Liu P., Cheng H., Roberts T.M., Zhao J.J. (2009). Targeting the phosphoinositide 3-kinase pathway in cancer. Nat. Rev. Drug Discov..

[306] Molina R.J., Adjei A.A. (2006). The Ras/Raf/MAPK pathway. J. Thorac. Oncol..

[307] Dhillon A.S., Hagan S., Rath O., Kolch W. (2007). MAP kinase signalling pathways in cancer. Oncogene.

[308] Klymchenko A.S., Kreder R. (2014). Fluorescent Probes for Lipid Rafts: From Model Membranes to Living Cells. Chem. Biol..

[309] Lingwood D., Simons K. (2007). Detergent resistance as a tool in membrane research. Nat. Protoc..

[310] Sajesh B.V., Guppy B.J., McManus K.J. (2013). Synthetic Genetic Targeting of Genome Instability in Cancer. Cancers.

[311] Paul J.M., Templeton S.D., Baharani A., Freywald A., Vizeacoumar F.J. (2014). Building high-resolution synthetic lethal networks: A ‘Google map’ of the cancer cell. Trends Mol. Med..

[312] Redmann M., Benavides G.A., Berryhill T.F., Wani W.Y., Ouyang X., Johnson M.S., Ravi S., Barnes S., Darley-Usmar V., Zhang J. (2017). Inhibition of autophagy with bafilomycin and chloroquine decreases mitochondrial quality and bioenergetic function in primary neurons. Redox Biol..

[313] NIH Clinical Trials Using Hydroxychloroquine. https://www.cancer.gov/about-cancer/treatment/clinical-trials/intervention/hydroxychloroquine.

[314] Briceño E., Reyes S., Sotelo J. (2003). Therapy of glioblastoma multiforme improved by the antimutagenic chloroquine. Neurosurg. Focus.

[315] Sotelo J., Briceno E., Lopez-Gonzalez M.A. (2006). Adding chloroquine to conventional treatment for glioblastoma multiforme: A randomized, double-blind, placebo-controlled trial. Ann. Intern. Med..

[316] Briceño E., Calderon A., Sotelo J. (2007). Institutional experience with chloroquine as an adjuvant to the therapy for glioblastoma multiforme. Surg. Neurol..

[317] Rojas-Puentes L.L., Gonzalez-Pinedo M., Crismatt A., Ortega-Gomez A., Gamboa-Vignolle C., Nuñez-Gomez R., Dorantes-Gallareta Y., Arce-Salinas C., Arrieta O. (2013). Phase II randomized, double-blind, placebo-controlled study of whole-brain irradiation with concomitant chloroquine for brain metastases. Radiat. Oncol..

[318] Eldredge H.B., DeNittis A., DuHadaway J.B., Chernick M., Metz R., Prendergast G.C. (2013). Concurrent whole brain radiotherapy and short-course chloroquine in patients with brain metastases: A pilot trial. J. Radiat. Oncol..

[319] Kyle R.A., Seligman B.R., Wallace H.J., Silver R.T., Glidewell O., Holland J.F. (1975). Mutiple myeloma resistant to melphalan (NSC-8806) treated with cyclophosphamide (NSC-26271), prednisone (NSC-10023), and chloroquine (NSC-187208). Cancer Chemother. Rep..

[320] King M.A., Ganley I., Flemington V. (2016). Inhibition of cholesterol metabolism underlies synergy between mTOR pathway inhibition and chloroquine in bladder cancer cells. Oncogene.

[321] Hsu S.P., Kuo J., Chiang H.-C., Wang H.-E., Wang Y.-S., Huang C.-C., Huang Y.-C., Chi M.-S., Mehta M.P., Chi K.-H. (2018). Temozolomide, sirolimus and chloroquine is a new therapeutic combination that synergizes to disrupt lysosomal function and cholesterol homeostasis in GBM cells. Oncotarget.

[322] Chude C.I., Amaravadi R.K. (2017). Targeting Autophagy in Cancer: Update on Clinical Trials and Novel Inhibitors. Int. J. Mol. Sci..

[323] Jones E.S., Jomary C. (2002). Clusterin. Int. J. Biochem. Cell Biol..

